# Self‐Driving Underwater “Aerofluidics”

**DOI:** 10.1002/advs.202301175

**Published:** 2023-04-28

**Authors:** Jiale Yong, Yubin Peng, Xiuwen Wang, Jiawen Li, Yanlei Hu, Jiaru Chu, Dong Wu

**Affiliations:** ^1^ CAS Key Laboratory of Mechanical Behavior and Design of Materials Key Laboratory of Precision Scientific Instrumentation of Anhui Higher Education Institutes Department of Precision Machinery and Precision Instrumentation University of Science and Technology of China Hefei 230027 P. R. China

**Keywords:** femtosecond laser, gas transportation, Laplace pressure, superhydrophobicity, underwater aerofluidics

## Abstract

Here, the concept of “aerofluidics,” in which a system uses microchannels to transport and manipulate trace gases at the microscopic scale to build a highly versatile integrated system based on gas‒gas or gas‒liquid microinteractions is proposed. A kind of underwater aerofluidic architecture is designed using superhydrophobic surface microgrooves written by a femtosecond laser. In the aqueous medium, a hollow microchannel is formed between the superhydrophobic microgrooves and the water environment, which allows gas to flow freely underwater for aerofluidic devices. Driven by Laplace pressure, gas can be self‐transported along various complex patterned paths, curved surfaces, and even across different aerofluidic devices, with an ultralong transportation distance of more than 1 m. The width of the superhydrophobic microchannels of the designed aerofluidic devices is only ≈42.1 µm, enabling the aerofluidic system to achieve accurate gas transportation and control. With the advantages of flexible self‐driving gas transportation and ultralong transportation distance, the underwater aerofluidic devices can realize a series of gas control functions, such as gas merging, gas aggregation, gas splitting, gas arrays, gas‒gas microreactions, and gas‒liquid microreactions. It is believed that underwater aerofluidic technology can have significant applications in gas‐involved microanalysis, microdetection, biomedical engineering, sensors, and environmental protection.

## Graphical abstract

Underwater aerofluidics is proposed and is realized by using superhydrophobic microgrooves to transport gas in an aqueous medium. Driven by Laplace pressure, gas can be self‐transported along various complex patterned paths. Underwater aerofluidic devices can realize a series of gas control functions, such as gas merging, gas aggregation, gas splitting, gas array, gas‒gas microreactions, and gas‒liquid microreactions.

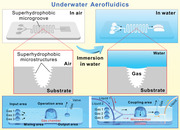

## 1 Introduction

Microfluidics has become an essential subject in modern science and technology, in which a system uses microchannels with a size of only tens to hundreds of microns to process or manipulate tiny fluids (nanoliters to attoliters in volume).^[^ ^1^, ^2^, ^3^ ^]^ The characteristics of miniaturization and integration enable microfluidic chips to perform a range of complex microprocess and micromanipulation, which are challenging to accomplish with traditional large‐scale test and analysis instruments.^[^ ^4^, ^5^, ^6^ ^]^ Over the past decades, microfluidic systems have been widely applied in chemical and biological analysis (e.g., genomic and proteomic research), cellular manipulation and detection, medical and health, high‐throughput pharmaceutical screening, and integrated optics due to significantly reduced chemical consumption, extremely accurate fluidic operations, and considerably accelerated reaction rates.^[^ ^7^, ^8^, ^9^ ^]^ Conventional microfluidic technology mainly focuses on processing and manipulating liquids; nevertheless, little attention is given to gases.

The transportation and manipulation of trace gases is a technology with great potential since many chemical reactions, analyses, and detection objects involve gases.^[^ ^10^, ^11^, ^12^, ^13^ ^]^ Spontaneous transport of bubbles underwater has been widely realized through topographic modulation of surface wetting gradients (such as superhydrophobic cones,^[^ ^14^, ^15^ ^]^ superhydrophobic wedge‐shaped structures,^[^ ^16^ ^]^ lubricated slippery cones,^[^ ^17^ ^]^ and slippery wedge‐shaped structures^[^ ^18^, ^19^ ^]^) that break the asymmetric contact line. However, gas transport based on these strategies is limited by short transport distances due to the fundamental tradeoff of hydrodynamics, and gases can only be manipulated macroscopically as a whole.^[^ ^20^ ^]^ Some stimulus‐driven methods (such as magnetic‐responsive^[^ ^21^, ^22^ ^]^ and photothermal‐responsive^[^ ^23^ ^]^) can theoretically move bubbles to arbitrary distances, but these transport methods are limited to complex substrate components and require continuous drive energy input. Most importantly, none of these methods can achieve microscopic precise transport and control of gas. Like microfluidics, tiny volumes of gas, from nanoliters to attoliters, can also be transported and manipulated through microchannels. Such a multifunctional system can be defined as “aerofluidics,” which manipulates trace gases at the microscopic scale to build highly versatile integrated systems based on gas‒gas or gas‒liquid microinteractions. Although Yong et al. preliminarily realized the gas transportation of aerofluidics in previous work,^[^ ^24^ ^]^ the basic principle and underlying mechanism of aerofluidics are still not well understood, and the functions and applications that aerofluidics can achieve have not been explored. It is believed that aerofluidics will have pioneering applications in gas‐involved microanalysis, microdetections, biomedical engineering, sensors, and environmental protection. However, achieving the transport of trace gas without the preparation of a closed channel inside solid substrates and without external energy consumption is still a challenge for aerofluidic systems.

Here, we propose a strategy to realize underwater aerofluidics using superhydrophobic microgrooves. Superhydrophobic microgrooves were directly written on a polydimethylsiloxane (PDMS) substrate by a femtosecond laser to connect the preprepared superhydrophobic inlet and outlet dots. When the aerofluidic device is immersed in water and a gas bubble is dispensed to the inlet dot, the gas can flow spontaneously to the outlet dot along the superhydrophobic microgroove, self‐driven by the Laplace pressure. The influences of the inlet and outlet area, the dimensions (i.e., length, depth, and width) of the microgroove, and the inclination angle on the gas transportation performance of underwater aerofluidic devices are systemically investigated. Diverse functions and applications, such as gas merging, gas aggregation, gas splitting, gas arrays, and bubble‐based gas‒gas and gas‒liquid microreactions, are demonstrated using the designed underwater aerofluidic devices. Potential strategies for functionalizing underwater aerofluidic devices and coupling aerofluidic devices with other devices are also proposed.

## 2 Results and Discussion

### 2.1 Concept and Mechanism of Underwater Aerofluidics

**Figure 1:**
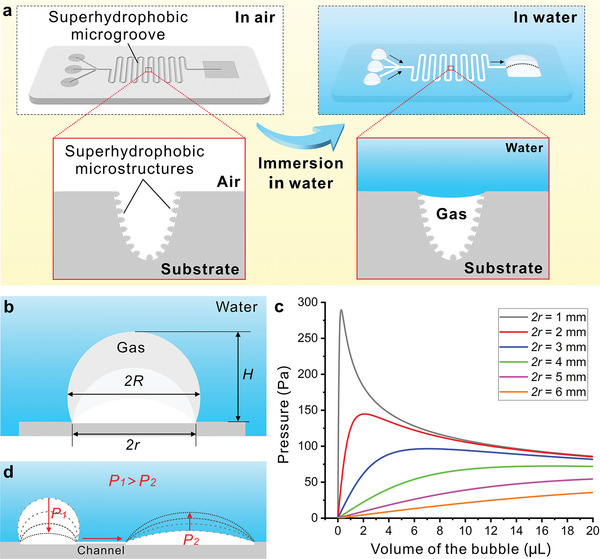
Concept of “underwater aerofluidics.” a) Schematic diagram of achieving underwater aerofluidics based on superhydrophobic surface microgrooves. b) Illustration of bubble shapes confined to a fixed circular base region in water. c) Pressure‒volume curves of underwater bubbles within the circular base dots (gas reservoirs) with the dot diameters (2*r*) ranging from 1 to 6 mm. d) Underwater gas transport model on surface aerofluidics: the gas in bubbles with higher internal pressure (*P*_1_) flows spontaneously to the low‐pressure (*P*_2_) dot along the Laplace pressure gradient (*P*_1_→*P*_2_).

Gas transportation is the essential technical support of aerofluidics. **Figure** **1**a illustrates the mechanism of achieving aerofluidics in an aqueous medium (called “underwater aerofluidics”) based on superhydrophobic surface microgrooves. Superhydrophobic open microgrooves are prepared on the surface of the chip substrate, connecting the inlet and outlet dots. The inner wall of the microgrooves is coated with superhydrophobic micro/nanostructures (left inset of Figure 1a). As the chip is immersed in water, the chip surface is covered by water. Water only wets the flat area, but it is unable to penetrate the superhydrophobic microgroove because the superhydrophobic microstructures greatly repel water (i.e., Cassie wetting state).^[^ ^25^, ^26^, ^27^ ^]^ As a result, a hollow closed channel forms between the substrate and the water environment (right inset of Figure 1a). This microchannel allows gas to flow through freely, thus endowing the chip with an aerofluidic function in a liquid medium.

The differential pressure across the gas‒liquid interface (known as the Laplace pressure) can be utilized as a passive pumping source for gas transport on underwater aerofluidic devices.^[^ ^24^, ^28^, ^29^ ^]^ Laplace pressure intrinsically exists in small bubbles, and the smaller the bubble is, the greater the pressure.^[^ ^24^, ^28^, ^29^, ^30^ ^]^ As shown in Figure 1b, when a small gas bubble is confined to a fixed base region (taking a circle as an example) on a solid surface in water, the shape of the bubble only depends on the size of the bottom dot and the gas volume (a bubble can be considered to be nearly spherical when its diameter is less than the capillary length).^[^ ^31^ ^]^ The internal Laplace pressure (*P*_L_) of the settling bubble under a certain gas volume (*V*) can be mathematically calculated as^[^ ^28^, ^31^, ^32^ ^]^

(1) $$\left\{\begin{array}{c} P_{L}=\frac{2\gamma }{R}=\frac{4H\gamma }{H^{2}+r^{2}}\\ V=\frac{\pi }{6}\left(H^{3}+3Hr^{2}\right) \end{array}\right.$$

where *γ* is the interfacial free energy between water and gas, *R* is the radius of curvature of the bubble, *H* is the height of the underwater bubble, and *r* is the radius of the bottom dot. Figure 1c plots the pressure‒volume curves of underwater bubbles within the circular base dot (gas reservoirs) with the dot diameters (2*r*) ranging from 1 to 6 mm. This diagram shows the Laplace pressure inside the bubble in relation to the diameter of the fixed bottom and the volume of the bubble. For an individual bottom dot, the internal pressure increases with the volume of the gas until it reaches a maximum value when a semispherical bubble shape is formed (*H* = *r*). It can also be seen that for a given bubble volume, the internal pressure of the settling bubble with a smaller bottom dot is consistently higher than that with a larger bottom dot, so there are pressure differences between gases on different base dots.^[^ ^31^ ^]^ As shown in Figure 1d, if bubbles on two base dots of different sizes are connected through an aerofluidic channel in a liquid medium, gas with higher internal pressure (*P*_1_) will flow along the Laplace pressure gradient to the low‐pressure (*P*_2_) dot until a hydrodynamic equilibrium (i.e., an equalized pressure level) is established. Therefore, the Laplace pressure of the small underwater bubble can provide the driving force of gas transport for underwater aerofluidic devices.

### 2.2 Preparation of the Underwater Aerofluidic Devices

**Figure 2:**
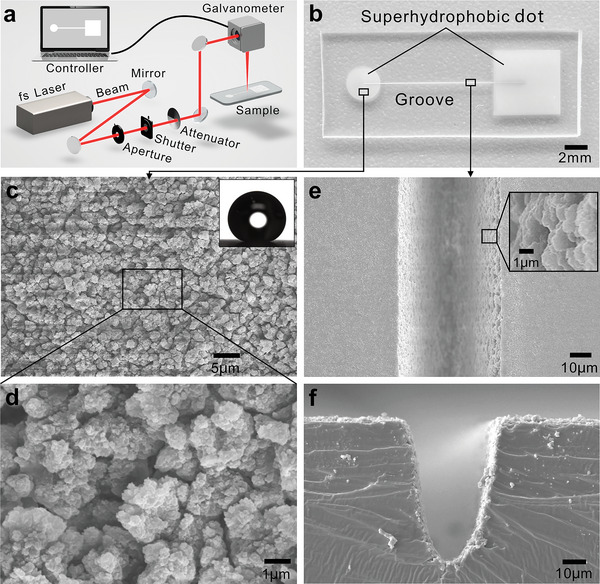
Designed underwater aerofluidic device. a) Femtosecond laser processing system for writing underwater aerofluidic patterns on a solid substrate. b) Photograph of the as‐prepared structure of the aerofluidic device on a PDMS substrate in which a laser‐written straight superhydrophobic microgroove connects two different superhydrophobic regions/dots. c,d) SEM images of the hierarchical microstructure on the laser‐ablated PDMS surface. The inset shows the profile of a water droplet on the structured surface with a CA of 155.1 ± 0.6°. e,f) SEM images of the laser‐written microgroove on the PDMS substrate: e) top view and f) profile of the section.

The most straightforward architecture (Figure S1a, Supporting Information) is designed to verify whether a superhydrophobic microgroove can be used to transport gas underwater. The verification process is schematically depicted in Figure S1b (Supporting Information). In the designed architecture, a straight superhydrophobic microgroove directly connects two different superhydrophobic dot patterns (as gas reservoirs). Such a structure can be easily prepared by femtosecond laser processing, which is an effective tool for designing the special surface wettability of solid materials.^[^ ^33^, ^34^, ^35^, ^36^ ^]^ **Figure** **2**a illustrates the femtosecond laser processing system for preparing underwater aerofluidic devices. The laser beam is guided into a high‐speed scanning galvanometer and focused onto the substrate surface by an *f‐θ* lens. PDMS is adopted as the substrate because it is the most widely used material in the microfluidic field and is inherently hydrophobic with an intrinsic water contact angle (CA) of 111.3 ± 1.8° (Figure S2a, Supporting Information). Figure 2b shows the as‐prepared aerofluidic device, in which the laser‐structured two dots (the small circular region acts as the inlet dot, and the large square region serves as the outlet dots) are connected by a laser‐written microgroove.

Figure 2c,d shows scanning electron microscopy (SEM) images of the PDMS surface ablated by a femtosecond laser at a laser power of 300 mW, scanning speed of 5 mm s^−1^, and scanning interval (*Λ*) of 25 µm. The ablated surface is uniformly covered with coral‐like hierarchical micro/nanostructures. Similar to a lotus leaf, the synergistic effect between the inherently hydrophobic chemistry of the PDMS substrate and the hierarchical microstructures makes the laser‐ablated zone have remarkable superhydrophobicity with a water CA of 155.1 ± 0.6° (inset of Figure 2c and Figure S2b in the Supporting Information).^[^ ^37^, ^38^ ^]^ The adhesive force between water and the laser‐structured surface is measured to be as low as 9.8 ± 2.0 µN (Figure S2c, Supporting Information), revealing that the superhydrophobic microstructure shows ultralow adhesion to water. The water droplets can easily roll away at a sliding angle (SA) of 1.4 ± 0.6° (Figure S2d and Movie S1 in the Supporting Information). When a free‐falling water droplet impacts the superhydrophobic structure, it can rebound more than 11 times (Figure S2e and Movie S1, Supporting Information). The ultralow adhesion reveals that the water droplet is in the Cassie wetting state on the superhydrophobic surface, i.e., water only contacts the top part of the laser‐induced microstructure (Figure S3a, Supporting Information).^[^ ^39^, ^40^, ^41^ ^]^ Figure S4 (Supporting Information) shows the influence of *Λ* on the wettability of the laser‐structured surface. With increasing *Λ*, the CA value decreases, while the SA value and the adhesion to water rise, indicating that the superhydrophobic property gradually weakens with increasing *Λ*. To balance the excellent superhydrophobicity and processing efficiency, all the superhydrophobic areas in our experiment were prepared at *Λ* = 25 µm. The femtosecond laser‐induced superhydrophobic microstructure also changes the wettability of the PDMS surfaces to underwater gas. The air bubble on the untreated PDMS substrate has a static CA of 96.8 ± 4.3°, advancing CA of 105.0 ± 2.3°, and receding CA of 60.1 ± 3.6° in water (Figure S5a–c, Supporting Information). For the superhydrophobic surface, once a bubble contacts the laser‐ablated area, the gas will spread out on the surface immediately (Figure S5d and Movie S2, Supporting Information), indicating that the PDMS surface becomes underwater superaerophilic after laser treatment. Figure 2e,f shows the morphology of the femtosecond laser‐written microgroove with ablation repetition times of 5 (at a laser power of 300 mW and a scanning speed of 5 mm s^−1^). The resultant groove has a width of 42.1 ± 2.6 µm and a depth of 70.4 ± 3.0 µm. The inner wall of the microgroove is entirely covered by the superhydrophobic microstructure induced by laser treatment.

### 2.3 Self‐Driving Gas Transportation

**Figure 3:**
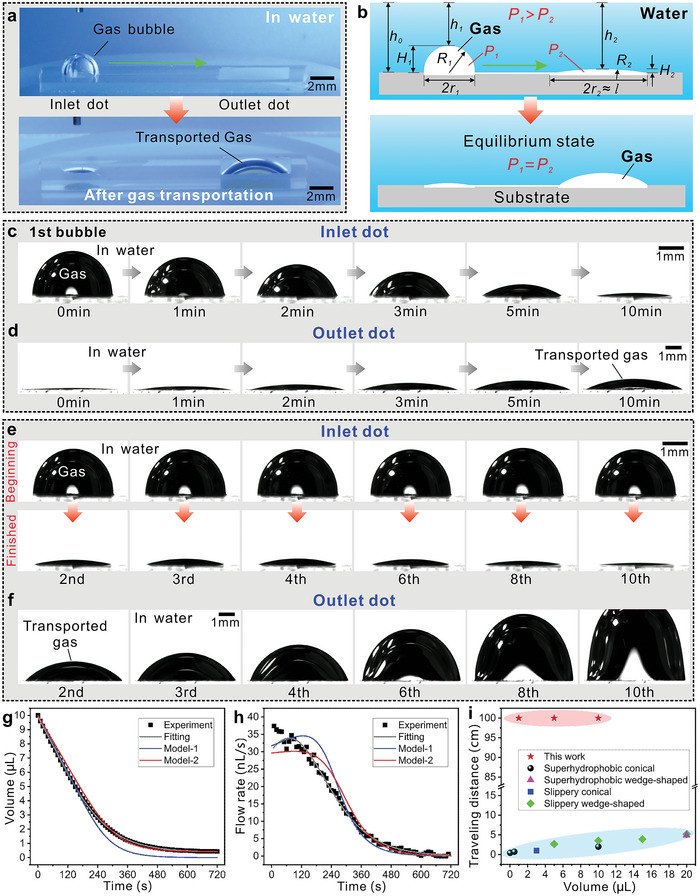
Self‐driving gas transportation of the underwater aerofluidic device. a) Inputting a 10 µL gas bubble into the inlet dot of the aerofluidic device in water (upper image) and the final state after the gas flows to the outlet dot (bottom image). b) Schematic diagram of the mechanism of spontaneous gas transportation driven by the Laplace pressure. c) Time‐lapse sequence of the shape variation of the gas at the inlet dot with time. d) Time‐lapse sequence of the bulging process of gas at the square outlet dot with time. e) Shape change of the gas bubbles at the inlet dot by continuously inputting air bubbles (one by one) into the aerofluidic system. The top images show the gas profiles before transportation, while the bottom images are the gas profiles at the complete end of the transportation process. f) The increased volume of the gas bulge at the outlet dot with continuously inputting air bubbles into the aerofluidic system. g) The volume change of the gas at the inlet dot with time during transportation. h) The gas flow rate with time during the transportation process. Model‐1 simply assumes that the area of the outlet dot is larger enough than that of the inlet dot, and Model‐2 approximately assumes that the gas bulge at the outlet dot has a spherical surface. i) Comparison of the gas traveling distance of the present underwater aerofluidic devices to other reported superwetting gradient structures, including superhydrophobic conical structures,^[^ ^14^, ^15^ ^]^ superhydrophobic wedge‐shaped structures,^[^ ^16^ ^]^ slippery conical structures,^[^ ^17^ ^]^ and slippery wedge‐shaped structures.^[^ ^18^, ^19^ ^]^

The as‐prepared underwater aerofluidic device works in an aqueous environment. When the device is dipped into water, water cannot wet the inlet dot, outlet dot, and connecting microgroove because of the excellent water repellence of the laser‐induced superhydrophobic microstructures. Similar to a lotus leaf in water, a silvery mirror‐like reflection (the so‐called “silver mirror effect”) appears in these areas due to the existence of trapped air (Figure S6, Supporting Information).^[^ ^42^ ^]^ Since water cannot penetrate the superhydrophobic microgroove, a hollow microchannel forms between the microgroove and the water cover (Figure S3b, Supporting Information). As shown in **Figure** **3**a, when a gas bubble with a volume of 10 µL is input into the inlet dot of the device in water, the gas is confined to the superhydrophobic/superaerophilic circular area, forming a hemispherical bubble. Figure 3c and Figure S7 and Movie S3 (Supporting Information) show the shape variation of the gas at the inlet dot as a function of time. The volume of the gas bubble decreases gradually with time. At the same time, the gas appears and swells in the outlet dot, and the volume of this gas bulge increases with time (Figure 3d and Movie S3 in the Supporting Information). The result demonstrates that the gas is successfully transported from the inlet to the outlet dot. At the end of the transportation process, most of the gas flows to the outlet dot (Figure 3a). Figure 3g records the variation trend of the gas volume with time at the inlet dot during transportation. At first, the gas flows fast and then gradually slows down. The gas flow rate also decreases with time (Figure 3h). When the transportation of the first bubble is complete, if the next bubble is further added to the inlet dot, the gas in this bubble can also flow to the outlet dot. Figure 3e and Movie S4 (Supporting Information) show the result of the continuous input of air bubbles (one by one) to the underwater aerofluidic device. All the gas can be transported to the outlet dot, with the volume of the gas bulge at the outlet dot continuously increasing (Figure 3f). The gas swells until the gas bulge is large enough that buoyancy will cause the gas bulge to detach from the outlet dot and rise to the water surface (Figure S8, Supporting Information). This process indicates that the underwater aerofluidic device can operate and transport gas continuously.

Interestingly, there is no external force input during the whole transportation process. The transportation process starts spontaneously, i.e., the system drives the gas flow. The underlying mechanism of such self‐driving gas transportation is schematically depicted in Figure 3b. The internal pressure of the gas bubble underwater is composed of the hydrostatic pressure and Laplace pressure.^[^ ^28^ ^]^ The amount of pressure (*P*) present within the gas bubble at the inlet dot and the air film/bulge at the outlet dot can be expressed as

(2) $$P_{1}=P_{0}+P_{h1}+P_{L1}$$

(3) $$P_{2}=P_{0}+P_{h2}+P_{L2}$$

where *P*_0_ is the atmospheric pressure, *P_h_* is the water pressure due to water depth, and *P*_L_ is the internal Laplace pressure. The subscripts 1 and 2 represent the parameters of gas at the inlet and outlet dots, respectively.

For the gas bubble at the inlet dot, *P_h_*_1_ and *P*_L1_ can be deduced as

(4) $$\left\{\begin{array}{c} P_{h1}=\rho gh_{1}=\rho g\left(h_{0}-H_{1}\right)\\ P_{L1}=\frac{2\gamma }{R_{1}} \end{array}\right.$$

In the same way, for the gas film/bulge at the outlet dot, *P_h_*_2_ and *P*_L2_ can be deduced as

(5) $$\left\{\begin{array}{c} P_{h2}=\rho gh_{2}=\rho g\left(h_{0}-H_{2}\right)\\ P_{L2}=\frac{2\gamma }{R_{2}} \end{array}\right.$$

where *ρ* is the density of water, *g* is the gravitational acceleration, *h*_1_ (*h*_2_) is the underwater depth of the gas surface at the inlet (outlet) dot, *h*_0_ is the distance from the water surface to the sample surface, *H*_1_ (*H*_2_) is the height of the underwater gas bubble at the inlet dot (the underwater gas film/bulge at the outlet dot), and *R*_1_ (*R*_2_) is the radius of curvature of the liquid/gas curved interface at the inlet dot (the outlet dot). The pressure difference, Δ*P*, between the gases at the inlet and outlet dots can thus be described as

(6) $$\begin{array}{ccc} & & \Delta P=P_{1}-P_{2}=\left(P_{h1}+P_{L1}\right)\;-\left(P_{h2}+P_{L2}\right)\\ & & =2\gamma \left(\frac{1}{R_{1}}-\frac{1}{R_{2}}\right)-\rho g\left(H_{1}-H_{2}\right) \end{array}$$

The deformation of the gas bubble at the inlet dot is negligible due to its small volume (the bound number $B_{0}=\frac{\rho gR_{1}^{2}}{3\gamma }\ll 1$), so the bubble can be approximately assumed to be perfectly spherical. The radius of curvature and the volume (*V*) of the gas at the inlet dot can be calculated using the following geometrical equations^[^ ^31^ ^]^

(7) $$\left\{\begin{array}{c} R_{1}=\frac{H_{1}^{2}+r_{1}^{2}}{2H_{1}}\\ V=\frac{\pi }{6}\left(H_{1}^{3}+3H_{1}r_{1}^{2}\right) \end{array}\right.$$

where *r*_1_ is the bottom radius of the circular inlet dot. When gas is just input into the inlet dot (before gas transportation), there is only the initial flat captured gas film at the outlet dot caused by the superhydrophobic microstructures. Thus, *H*_2_ and *R*_2_ are close to 0 and ∞, respectively. The initial pressure difference, Δ*P*_0_, can be simplified as

(8) $$\Delta P_{0}=\frac{2\gamma }{R_{1}}-\rho gH_{1}$$

The first term in this formula is the Young–Laplace pressure of the input gas bubble, which can be calculated as ≈95 Pa by combining it with Equation (7). The second term, the pressure caused by the height of the bubble, is only 10 Pa, which is far smaller than the internal Laplace pressure. Therefore, Δ*P*_0_ >> 0. The pressure gradient (mainly caused by the Laplace pressure) is enough to drive gas to flow from the inlet dot to the outlet dot along the underwater superhydrophobic microgroove until the system reaches an equilibrium state, resulting in the spontaneous gas transportation process (Figure 3b).

In the transportation process, the volume of the gas bubble at the inlet dot decreases, and gas gradually bulges from the outlet dot. The pressure difference also changes dynamically. We adopt two approximate methods to describe the evolution of the gas bubble at the inlet dot during gas transportation (see the Experimental Section for details).^[^ ^33^, ^37^, ^38^, ^43^ ^]^ The model values based on the deduced differential equations are added in Figure 3g,h to compare with the experimental values, as the Model‐1 line (simply assuming that the area of the outlet dot is larger enough than that of the inlet dot) and the Model‐2 line (approximately assuming that the gas bulge at the outlet dot has a spherical surface), respectively. The agreement between the experimental results and the theoretical prediction verifies that the Laplace pressure is the main driving force for our underwater aerofluidic devices.

### 2.4 Influencing Factors on Gas Transportation Performance

**Figure 4:**
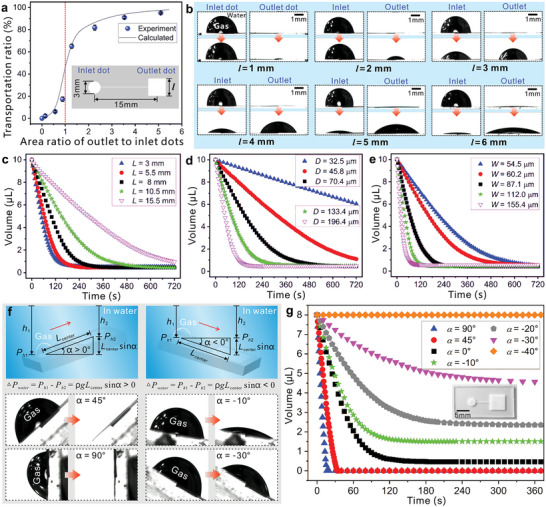
Influencing factors on the gas transportation performance of the underwater aerofluidic device. a) Relationship between the gas transportation ratio and the area ratio of the outlet to inlet dots. The inset shows the structure of the designed underwater aerofluidic device. b) The gas profile change (at the inlet dot and the outlet dot) before and after gas transportation, with *l* changing from 1 to 6 mm. The top images show the gas profiles before transport, while the bottom images are the gas profiles at the complete end of the transportation process. c–e) Influence of c) the length (at *D* = 70.4 µm and *W* = 42.1 µm), d) the depth (at *L* = 10.5 mm and *W* = ≈42.3 µm), and e) the width (at *L* = 10.5 mm and *D* = ≈41.4 µm) of the laser‐induced microgrooves on the gas transportation of the underwater aerofluidic devices. The overall trends of *L* = 15.5 mm in (c), *D* = 32.5 µm and *D* = 45.8 µm in (d), and *W* = 54.5 µm in (e) are provided in Figure S11 in the Supporting Information. f) Influence of the inclination angle (*α*) on the gas transportation performance of the underwater aerofluidic device. The first line schematically illustrates that the aerofluidic device is tilted upward (left image) with a positive *α* value or downward (right image) with a negative *α* value. The second and third lines show the profile change of an input gas bubble at the inlet dot before (left image) and after (right image) gas transportation at *α* values of 45°, 90°, −10°, and −30°. g) Volume change of the gas with time during gas transportation at different inclination angles. The inset shows the aerofluidic structure for measurement.

The sizes of the superhydrophobic inlet dot and the outlet dot play a crucial role in the gas transportation performance of the underwater aerofluidic device. To investigate this influencing factor, we fix the diameter (2*r*_1_ = 3 mm) of the circle at the inlet dot and change the side length (*l*) of the square area from 1 to 6 mm at the outlet dot (inset of **Figure** **4**a). The gas transportation capability of these aerofluidic devices is compared and summarized in Figure 4a,b and Movie S5 (Supporting Information). In the case of *l* = 1 mm, the area of the outlet dot is much smaller than that of the inlet dot. The area ratio of the outlet to inlet dot ($\eta_{\mathrm{area}}=\frac{S_{\mathrm{outlet}}}{S_{\mathrm{inlet}}}$, where *S*_outlet_ and *S*_inlet_ are the areas of the outlet dot and the inlet dot, respectively) is only ≈0.142. The gas is difficult to transport, and only a tiny amount of gas flows to the outlet dot. The gas transportation ratio ($\eta_{\mathrm{gas}}=\frac{V_{0}-V_{\mathrm{final}}}{V_{0}}\times 100\%$, *V*_0_ is the volume of input gas, and *V*_final_ is the volume of the residual gas remaining at the inlet dot after gas transportation) is as low as 2.2%. As *l* increases to 2 mm, the volume of gas reaching the outlet dot also increases, but *η*_gas_ is still far below 50%. When *l* increases to 3 mm, the area of the outlet dot is larger than that of the inlet dot (*η*_area_ > 1). In this case, *η*_gas_ is greater than 50%, indicating that more than half of the gas is transported. Further increasing *l* and *η*_area_ enables the transport of more gas. When *η*_area_ is increased to 5.1 (*l* = 6 mm), *η*_gas_ can reach 95.1%. Such a high value indicates that almost all the gas at the inlet dot successfully flows to the outlet dot along the superhydrophobic microgroove. It is demonstrated that the larger the area of the outlet dot, the more conducive it is to gas transport (Figure 4a). If we want to transport more than 95% of the input gas, the area ratio of the outlet dot to the inlet dot must be at least 5.

The width and depth of the superhydrophobic microgrooves for gas transport can be easily designed by laser processing parameters, as shown in Figure S9 (Supporting Information). Both the width and depth increase with increasing laser power and decrease with increasing scanning speed. The depth of the microgroove obviously increases with increasing ablation times, although the width is less affected by the number of ablation times. Figure 4c–e illustrates the influence of the length (*L*), depth (*D*), and width (*W*) of the laser‐induced connecting microgrooves on the gas transportation of underwater aerofluidic devices. The layout of the structure parameters (*L*, *D*, and *W*) is depicted in Figure S10 (Supporting Information). For a longer microgroove, the gas has to travel a long distance. The resistance to gas transport is more significant, so the gas flows more slowly (Figure 4c). The flow rate decreases with increasing *L* (Figure S12a, Supporting Information). As *L* increases from 3 to 15.5 mm, the initial gas flow rate decreases from 110.5 to 29 nL s^−1^. The gas transportation length can even extend to more than 1 m (see Section 2.5 for details). A deeper microgroove (Figure 4d) or a wider microgroove (Figure 4e) can speed up the gas transportation process because the increase in the depth and width will lead to an increase in the cross‐sectional area of the underwater microchannel. The flow rate increases linearly with the depth and width of the superhydrophobic microgrooves (Figure S12b,c, Supporting Information). For example, the initial flow rate increases from 2.5 to 111.0 nL s^−1^ as *D* increases from 32.5 to 196.4 µm, and it increases from 25.5 to 242.0 nL s^−1^ as *W* increases from 54.5 to 155.4 µm.

Tilt can also affect the self‐driving gas transportation process. The gas transport of the as‐prepared underwater aerofluidic device under different inclination angles (*α*) is investigated, as shown in Figure 4f. *α* is defined as positive for upward tilt and negative for downward tilt. In the case of *α* > 0°, when the device is tilted at 45° or even placed vertically (*α* = 90°), almost all the gas at the inlet dot can be transported to the outlet dot (left side of Figure 4f). In contrast, when the device is tilted at −10° and −30°, some gas remains at the inlet dot as the transportation process is completed (right side of Figure 4f). The gas transportation process no longer occurs when the device is tilted down at 40° (*α* = ‐40°). Figure 4g and Movie S6 (Supporting Information) show the gas volume changes (at the inlet dot) in the gas transportation process at different *α*. For a higher value of *α*, the gas is transported faster. The flow rate increases linearly with increasing *α* (Figure S13a, Supporting Information). As *α* increases from −30° to 0° and then to 90°, the initial gas flow rate increases from 27 to 129 and then to 572 nL s^−1^. On the other hand, with the increase in *α*, the volume of the residual gas at the inlet dot decreases, and more gas is transported to the outlet dot (Figure S13b, Supporting Information). The results demonstrate that the upward tilt of the underwater aerofluidic device is beneficial to gas transportation, while the downward tilt hinders gas transportation.

When the aerofluidic device is tilted in water, the inlet and outlet dots are at different water depths, so there is a pressure difference, △*P*_water_, between the two positions affected by water depth. As shown in the first line of Figure 4f, △*P*_water_ can be expressed as

(9) $$\Delta P_{\mathrm{water}}=P_{h1}-P_{h2}=\rho gh_{1}-\rho gh_{2}=\rho gL_{\mathrm{center}}\sin\alpha$$

where *L*_center_ is the center distance between the inlet dot and the outlet dot, and there is *h*_1_ − *h*_2_=*L*_center_sin *α* according to the geometric correspondence. It can be seen from this formula that when the device is tilted upward (*α* > 0°), △*P*_water_ is greater than 0 (left side of Figure 4f). This extra positive pressure facilitates gas flow from the inlet to the outlet dot. In contrast, when the device is tilted downward (*α* < 0°), △*P*_water_ is negative (right side of Figure 4f). This additional negative pressure will weaken the driving force in the self‐driving gas transportation process, hindering the efficient transport of gas.

### 2.5 Versatile Performance of the Underwater Aerofluidic Devices

**Figure 5:**
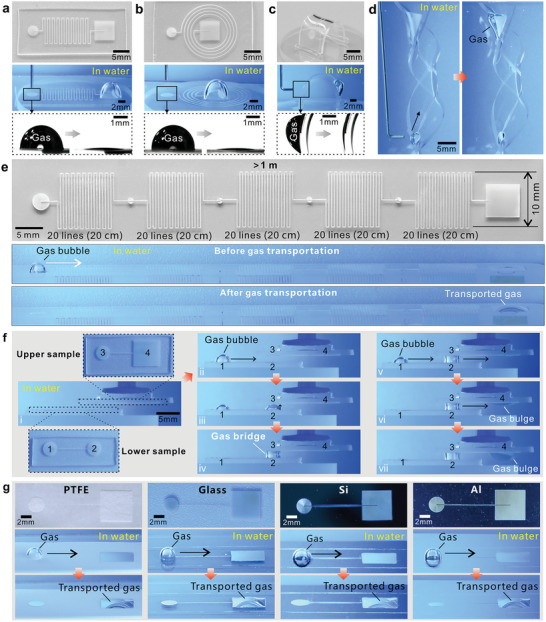
Versatile gas transportation by using the designed aerofluidic devices. a,b) Gas transportation on the complex underwater aerofluidic devices in which the inlet dot and outlet dot are connected by a) a continuous‐curve line and b) a spiral line, respectively. c) Gas transportation on the curved underwater aerofluidic device. The first line in (a–c) is the photos of the aerofluidic devices, the second line depicts that a certain amount of gas has been transported from the inlet dot to the outlet dot, and the third line shows the profile change of an inputting gas bubble. d) Gas transportation on a 3D twisted aerofluidic device in water. e) Gas transportation on an integrated aerofluidic device where five individual curved‐line components are connected in series. The first line shows the structure layout, and the second and third lines show the moment before and after gas transportation. The gas travels a total distance of more than 1 m. f) Gas transportation across different aerofluidic devices (on two substrates) by rationally utilizing the spatial location and constructing a connecting gas bridge. g) Self‐driving gas transportation on different substrates. The first line shows the structures of the aerofluidic devices, and the second and third lines show the moment before and after gas transportation.

Gas can be transported not only in straight superhydrophobic microgrooves but also in various complex paths. For example, **Figure** **5**a,b shows two aerofluidic devices whose inlet and outlet dots are connected by a continuous curve and a spiral line, respectively. As the gas bubbles are continuously inputted, the gas eventually flows to the outlet dots (Movie S7, Supporting Information), demonstrating that even the complex curved microgrooves still ensure the most basic gas transportation function of the aerofluidic devices. In addition to the flat surface, gas can also be transported along curved surfaces. As shown in Figure 5c, a simple aerofluidic device on a PDMS sheet is bent into a curved surface and submerged in water. When the gas is dispensed onto the inlet dot, the gas is able to cross the curved surface and successfully reach the outlet dot on the other side of the curved surface (Movie S7, Supporting Information). Even when the aerofluidic device is twisted into a 3D spiral shape, the gas can be transported normally (Figure 5d). The bendability enables the underwater aerofluidic device to be used as a flexible device to adapt to the more complex working environment. Interestingly, gas can pass from one side of the PDMS sheet to another side when the inlet and outlet dots are separated on either side of the sheet and connected through superhydrophobic through‐holes. Even antibuoyance (downward direction) gas penetration can be realized (Figure S14, Supporting Information), which makes it possible to integrate aerofluidic devices with other devices, such as the microfluidic system.

Individual underwater aerofluidic devices can be connected in series or parallel to integrate into a multifunctional system. By connecting the superhydrophobic microgrooves in series, the gas traveling distance can be gradually increased. In this way, we can study the gas transportation capacity of the aerofluidic devices (Figure S15 and Movie S8, Supporting Information). Figure 5e shows an integrated aerofluidic device containing five curved‐line components, each 20 cm long. The total length of the microgrooves in this system is greater than 1 m (*W* = ≈42.5 µm and *D* = ≈133.4 µm). Surprisingly, when a 10 µL gas bubble is dispensed onto the inlet dot, it will slowly shrink. The gas also swells in the outlet dot, and its volume increases gradually with time. The result demonstrates that the input gas successfully flows from the inlet dot to the outlet dot, although this transportation process takes a long time (Figure S16 and Movie S9, Supporting Information). The gas traveling distance (1 m) in the present aerofluidic device is 20 times longer than that of the previously reported superwetting geometric gradient‐shaped structures (e.g., superhydrophobic cone,^[^ ^14^, ^15^ ^]^ superhydrophobic wedge,^[^ ^16^ ^]^ lubricated slippery cone,^[^ ^17^ ^]^ and slippery wedge^[^ ^18^, ^19^ ^]^), where the macroscopic gas transportation is usually at the centimeter level (Figure 3i).

Spatial location can be cleverly used to achieve the association between different aerofluidic devices (on different substrates). Figure 5f and Movie S10 (Supporting Information) illustrate a strategy for transporting gas across different substrates in an integrated aerofluidic system. A bottom device (containing superhydrophobic Region 1 and Region 2) and a top device (containing superhydrophobic Region 3 and Region 4) are placed face to face in the water, with Region 3 directly above Region 2 (Step i). When a gas bubble is added to Region 1, a hemispherical bubble is formed here. Since the curvature radius of the bubble is much smaller than that of the flat trapped air film at superhydrophobic Region 2, the Laplace pressure inside the bubble is greater than that of the trapped air film (Equation (6)). The Laplace pressure difference drives the gas to flow to Region 2 (Step ii), and a gas bulge will swell from Region 2. The height of the gas bulge gradually increases with continuous gas transfer until its top reaches the trapped gas film above superhydrophobic Region 3 (Step iii). At this moment, the gas inside the gas bulge at Region 2 and the gas inside the trapped air film at Region 3 merge. A hollow gas bridge is therefore constructed between Region 2 and Region 3 (Step iv), allowing gas to freely pass through it to connect the two underwater aerofluidic devices. Thus, Region 1 and Region 4 are connected by the superhydrophobic microgroove (between Region 1 and Region 2) on the bottom substrate, the formed gas bridge, and the superhydrophobic microgroove (between Region 3 and Region 4) on the top substrate. The internal Laplace pressure of the input gas at Region 1 is greater than that at Region 4 because Region 4 has the largest area. In addition, the water depth of Region 4 is less than that of Region 1, which also causes a pressure difference. Both the Laplace pressure difference and the depth pressure difference tend to flow gas from Region 1 to Region 4, according to Equations (6) and (9). When gas is continuously imported into Region 1, the gas is transported to Region 3 (Step v) through the gas bridge and further transferred to Region 4 (Steps vi and vii), which indicates that the gas is successfully transported across different aerofluidic substrates. By constructing connecting gas bridges, gas transportation can be realized across more independent aerofluidic devices.

The superhydrophobicity of the laser‐induced PDMS microstructures is very stable (Figure S17a, Supporting Information), which allows the as‐prepared aerofluidic device to work in various harsh environments (Movie S11, Supporting Information). For example, the underwater gas can be normally transported on the aerofluidic device even though the device is previously heated at 200 °C for 6 h (Figure S17b, Supporting Information) or irradiated by UV light for 6 h (Figure S17c, Supporting Information). The device also maintains the gas transportation capacity when it is immersed in HCl acid solution (pH = 2), NaOH alkali solution (pH = 12), and 10% NaCl salt solution (Figure S17d–f, Supporting Information). In addition to the PDMS surface, the underwater aerofluidic structure can also be prepared on a variety of substrates by femtosecond laser processing. For example, Figure 5g shows simple underwater aerofluidic systems designed on polytetrafluoroethylene (PTFE), glass, silicon, and aluminum substrates. The femtosecond laser‐induced micro/nanostructures on these substrates show excellent superhydrophobicity (Figure S18, Supporting Information). Underwater self‐driving gas transportation can be achieved on these substrate materials using superhydrophobic surface microgrooves (Figure 5g and Movie S12 in the Supporting Information).

### 2.6 Diverse Functions and Applications of Gas Manipulation

**Figure 6:**
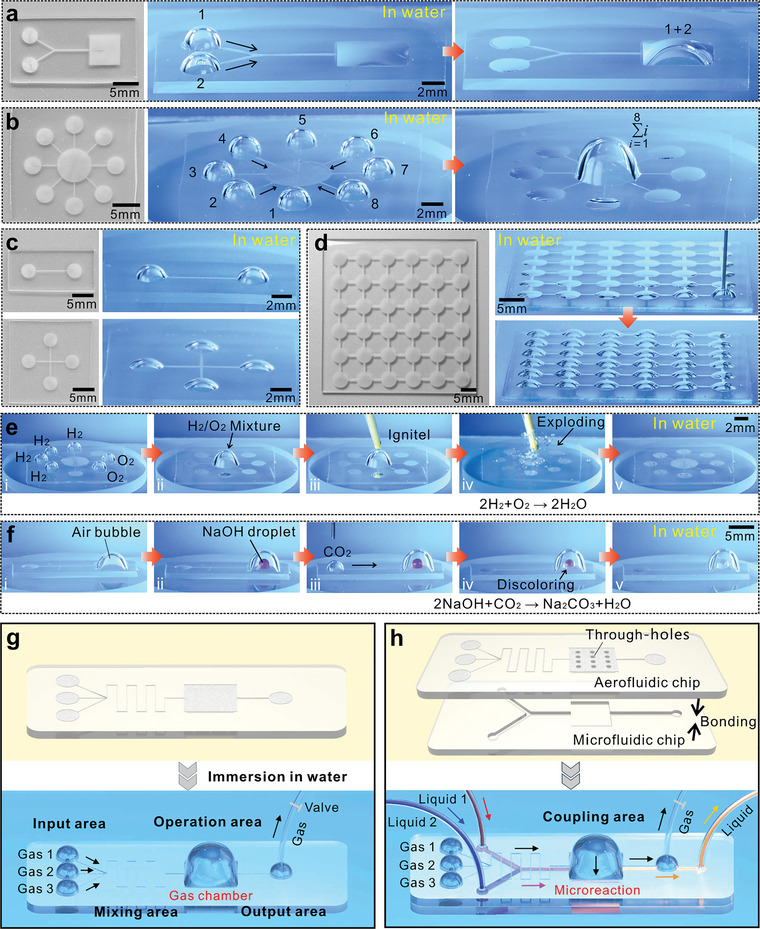
Various gas‐involved functions and applications of designed underwater aerofluidic devices. a) Merging of two gas bubbles. b) Aggregation of multiple gas bubbles. The left images in (a,b) show the structures of the aerofluidic devices, the middle images show the moment that bubbles are inputted into the inlet dots, and the right images are the states after gas merging or aggregation. c) Splitting a gas bubble into two (first‐line images) and four (second‐line images) equal parts. The left images show the aerofluidic structures for gas splitting, and the right images show that a bubble is split equally in the circles. d) Formation of an array of gas bubbles using the aerofluidic device consisting of 6 × 6 interconnected superhydrophobic circular regions. e) Microreaction between the H_2_ gas and the O_2_ gas on the underwater aerofluidic device. f) Microreaction between the CO_2_ gas and the alkaline NaOH solution consisting of phenolphthalein reagent on the underwater aerofluidic device. g,h) Potential strategies for functionalizing underwater aerofluidic devices and coupling with other devices: g) design of different basic functional areas on an underwater aerofluidic chip, h) integration between underwater aerofluidic chip and microfluidic chip through superhydrophobic connecting holes. The upper images show the structural layout of the designed devices in air, while the bottom images show how the aerofluidic devices work in water.

Flexible gas transportation and ultralong transportation distance allow underwater aerofluidic devices to perform various gas manipulations in a liquid medium. **Figure** **6**a shows a device that can realize the gas merging function. Two circular inlet regions are connected to a larger square outlet region by a “Y”‐shaped superhydrophobic microgroove. When the two bubbles are dispensed into the two inlet dots, the gas in the two bubbles can be transported to the outlet dot and eventually coalesce (Movie S13, Supporting Information). The gas merging process can be extended to merge more gas bubbles, enabling the aggregation of multiple bubbles. For example, Figure 6b shows a gas aggregation device in which eight inlet dots are connected to a larger collecting domain. The gas flows from the eight inlet dots to the collecting domain simultaneously (Movie S13, Supporting Information). The functions of gas merging and gas aggregation allow the mixing of trace amounts of different gases on demand.

As an inverse process of gas merging, gas splitting has also attracted wide attention because of its important applications in fermentation, heat transfer, water treatment, energy harvesting, etc.^[^ ^30^ ^]^ Interestingly, if many superhydrophobic regions of the same shape and size are interconnected (without a larger collecting domain), the designed aerofluidic device can easily achieve the gas splitting function (i.e., approximate equipartition of a gas bubble). The first line of Figure 6c shows the simplest demonstration device where a laser‐written superhydrophobic microgroove connects two superhydrophobic circular regions. When a gas bubble is dispensed into any circular area, half of the gas can travel to the other circular dot along the connecting line, so the original bubble is split equally into two circles (Figure S19a, Supporting Information). In addition to bisecting, the gas can also be divided into more parts. For example, devices consisting of three and four interconnected circular regions can divide a gas bubble into three or four equal parts as the gas bubble is released onto any circular region (Figure 6c and Figure S19b,c in the Supporting Information). As a result, a gas bubble is evenly split into smaller volumes of separate bubbles. This method allows bubbles to be split on demand by connecting a set number of superhydrophobic regions. Compared to the reported buoyancy‐based bubble splitting method,^[^ ^30^ ^]^ in which the small bubbles after splitting quickly rise to the water surface, the gas splitting method based on underwater aerofluidics has better operability because the split gas can be fixed on a solid surface and remain in the liquid for a long time. Furthermore, bubbles can also be arrayed by connecting more superhydrophobic circles. Figure 6d shows an array of 6 × 6 interconnected superhydrophobic circular dots. When the device is immersed in water and gas is continuously injected into one dot, bubbles swell from other circles and form an array of gases (Movie S14, Supporting Information). It has been reported that such a gas array can significantly improve the efficiency of gas‒liquid interactions (e.g., in the treatment of industrial and domestic wastewater) because it can not only prolong the gas retention time but also enlarge the gas/liquid contact area in aqueous media.^[^ ^44^ ^]^

As a green, low‐cost, and highly efficient chemical reaction carrier, droplet‐based microreactors have attracted increasing attention in surface microfluidics.^[^ ^45^, ^46^ ^]^ However, there are few reports on microreactions between gases due to the difficulty of bubble manipulation, i.e., the bubble floats quickly and easily deforms. With the help of the aerofluidic device, we can realize underwater microreactions between different gases with a volume of only a few microliters. A proof‐of‐concept experiment is carried out by using a simple and classic explosive reaction. As shown in Figure 6e, four hydrogen bubbles and two oxygen bubbles that are inputted onto the aggregation‐functional aerofluidic device (Step i) can spontaneously flow to the collection domain and mix together (Step ii). When the mixed bubble composed of H_2_ and O_2_ gases is ignited by the piezoelectric ceramic electron flame (Step iii), an explosive microreaction (2H_2_ + O_2_ → 2H_2_O) occurs instantaneously (Step iv), as shown in Figure S20 and Movie S15 (Supporting Information). The volume of the mixing bubble shrinks dramatically because the chemical microreaction consumes H_2_ and O_2_ gases (Step v). The explosive reaction proves the feasibility of a bubble‐based microreactor of underwater aerofluidic devices.

In addition to gas‒gas reactions, the aerofluidic device can also achieve gas‒liquid microreactions. The classical color reaction is utilized to demonstrate this application, as shown in Figure 6f and Movie S16 (Supporting Information). A certain volume of air is previously injected into the underwater aerofluidic device in an aqueous environment, resulting in the formation of a large gas bulge at the outlet dot (Step i). The gas bulge acts as a reaction chamber. Then, a 7 µL sodium hydroxide droplet containing a certain amount of phenolphthalein reagent is dispensed on the outlet dot inside the gas bulge (Step ii). The droplet is purple at this moment. When the CO_2_ gas is inputted onto the inlet dot (Step iii), the gas will flow to the gas bulge (Step iv). Meanwhile, the color of the droplet gradually changes from purple to transparent as the acidic gas is continuously mixed in the gas bulge (Step v). The decolorization of phenolphthalein is caused by the decreased pH value, demonstrating that the acidic gas reacts with the alkaline solution (2NaOH + CO_2_ → Na_2_CO_3_ + H_2_O). Although the abovementioned gas‒gas and gas‒liquid microreaction systems are relatively simple, the concept of bubble‐based microreactors is of great significance in gas‐involved chemical analysis and detection.

In potential practical applications, aerofluidic devices need to be functionalized and divided into different functional areas. For example, Figure 6g depicts the most basic compositions of an underwater aerofluidic chip, mainly including input, mixing, operation, and output areas. Different kinds of gas can be input into this system at the input area. The gases will spontaneously flow to the core region along the curved connecting microgroove at the mixing area, and the mixing function is achieved. A large gas bulge (chamber) forms above the core region at the operation area with the largest superhydrophobic region. The excess gas is eventually discharged into the atmosphere through a thin tube that connects the small gas bulge at the output area. The gas chamber can be used as a micro‐operating environment, allowing the chip to perform a range of complex gas‐involved microprocess and micromanipulation within a small space. For example, if liquid droplets are introduced into the gas chamber, the operation between gas and liquid will become possible. The air chamber can also provide a microenvironment for the culture of microorganisms or tiny animals. The basic functions of the different areas of the functionalized aerofluidic device have been demonstrated separately in the abovementioned experiments (e.g., Figures 5 and 6a–f). The underwater aerofluidic chip can also be coupled with other microdevices, such as microfluidic devices, to achieve the interaction between liquid and gas. As shown in Figure 6h, the underwater aerofluidic chip and the liquid microfluidic chip are integrated by superhydrophobic connecting through‐holes. The microholes allow the gas on the aerofluidic chip to reach the bottom side of the chip (Figure S14, Supporting Information) and contact the underneath liquid flow in the microfluidic chip. A proof‐of‐concept demonstration of the interaction between the underwater aerofluidic system and the microfluidic system is shown in Figure S21 (Supporting Information). The aerofluidic device is also able to provide different gas environments for different areas of microfluidics. A gas‒liquid integrated system is thus realized, whose multiple functions are beyond a single microfluidic or aerofluidic system.

## 3 Conclusions

In conclusion, underwater aerofluidics is proposed and realized by using superhydrophobic microgrooves to transport gas in an aqueous medium. In water, gas can flow spontaneously from the inlet to the outlet dot of the aerofluidic device along the superhydrophobic microgroove without an external force input. The whole gas transportation process is self‐driven by the Laplace pressure. The width of the superhydrophobic microchannels of the designed aerofluidic devices is only ≈42.1 µm. Such a narrow microchannel allows the aerofluidic system to achieve accurate gas transportation and manipulation. The gas flow rate depends on the length, depth, and width of the connecting microgrooves, as well as the inclination angle. The flow rate of the aerofluidic devices can be designed from very slow to ultrafast (e.g., from 2.5 to 572 nL s^−1^ in this experiment) on demand. The aerofluidic device can even support an extremely long gas transportation distance of more than 1 m, which is tens of times longer than the previously reported superwetting geometric gradient‐shaped structures. Gas can be transported along various complex paths, curved surfaces, and even across different aerofluidic devices. Individual underwater aerofluidic devices can be connected in series or parallel to integrate into a multifunctional system. The advantages of flexible self‐driving gas transportation and ultralong transportation distance endow the designed underwater aerofluidic devices with a series of gas‐control functions, such as gas merging, gas aggregation, gas splitting, gas array, bubble‐based gas‒gas microreaction, and gas‒liquid microreaction. Self‐driving underwater aerofluidics aims to transport and manipulate trace gases at the microscopic scale to build highly versatile integrated systems, which has significant potential applications in gas‐involved microanalysis, microdetection, biomedical engineering, sensors, and environmental protection.

Unlike the previously reported macroscopic manipulation of bubbles (the overall movement of bubbles), the gas manipulation proposed here is a microscopic, continuous, and differential method. Aerofluidics enables precise and complex manipulation of trace gases (e.g., nanoliter) that cannot be achieved by those monolithic (as a whole) bubble transfer methods. In addition, femtosecond laser processing is a free and flexible machining method. The machining path can be precisely controlled so that a variety of complex aerofluidic patterns can be easily designed and prepared. The characteristics of femtosecond laser microfabrication provide infinite imagination space for preparing multifunctional aerofluidic devices.

## 4 Experimental Section

### Materials

Solid PDMS sheets with a thickness of ≈1 mm were prepared by mixing the prepolymer and curing agent (Sylgard 184, Dow Corning Corporation) with a volume ratio of 10:1 and heating at 80 °C for 4 h. PTFE and 1060 aluminum sheets were purchased from YuanDongLi Internet Corporation. Ordinary microscope slides (CITOTEST) were used as glass substrates. Silicon wafers were purchased from SILICON WAFER Corporation. 1*H*,1*H*,2*H*,2*H*‐Perfluorodecyltriethoxysilane (PFDTES) was purchased from Aladdin Industrial Corporation. Deionized water was used as the liquid environment. The main tested gas was directly obtained from the air. Other special gases (H_2_, O_2_, and CO_2_) were industrial‐grade pure gases.

### Femtosecond Laser Treatment

The femtosecond laser beam with a pulse duration of 104 fs, central wavelength of 800 nm, and repetition rate of 1 kHz was produced by a regenerative‐amplified Ti:sapphire femtosecond laser system (Legend Elite‐1K‐HE, Coherent). As shown in Figure 2a, the laser beam was guided into a computer‐controlled high‐speed galvanometer scanner (SCANLAB, Germany) and further focused on the sample surface through a telecentric *f‐θ* lens (focal length of 63 mm). The focused laser beam has a spot diameter of ≈20 µm. In the laser ablation process, the processing software controlled the scanning path (Samlight). The superhydrophobic microgrooves were directly written on the sample surface by femtosecond laser scanning, while the superhydrophobic regions were prepared based on the typical line‐by‐line laser scanning method at a laser power of 300 mW, scanning speed of 5 mm s^−1^, and scanning interval (*Λ*) of 25 µm. After laser ablation, the samples were cleaned ultrasonically with ethanol and deionized water for 10 min each.

### Superhydrophobic Treatment

PDMS and PTFE are inherently hydrophobic substrates. They directly exhibit superhydrophobicity after femtosecond laser processing. In contrast, glass, silicon, and aluminum are hydrophilic substrates. Their surface‐free energy needs further reduction after laser ablation to become superhydrophobic. The laser‐structured glass, silicon, and aluminum surfaces were hydrophobicated by the chemical vapor deposition (CVD) of fluorosilane reagent. The structured surfaces were fluorinated in a closed container (volume = 135 mL) with a 10 µL drop of PFDTES for 24 h at room temperature.

### Characterization

The morphology of the laser‐structured surfaces was observed by an SEM (GeminiSEM 500, Carl Zeiss). The wettabilities of the water droplets and underwater bubbles on the sample surfaces were investigated by a contact angle measurement (CA100C, Innuo). The ultrafast processes of droplet rebounding, underwater gas spreading, bubble detaching from the substrate, and the explosive reaction of the bubble‐based microreactor were recorded by a high‐speed camera (Chronos 2.1‐HD, Kron Technologies) at a frame rate of 1000 fps. The processes of self‐driving gas transportation on underwater aerofluidic devices were recorded by a digital camera (D7100, Nikon).

### Approximate Dynamic Analysis of the Gas Transportation Process

In the transportation process, the gas volume at the inlet and outlet dots changed with time. The pressure difference also changed dynamically as the gas volume changed. Here, two approximate methods were adopted to simplify the problem. In the first case, it was assumed that the area of the outlet dot is larger enough than that of the inlet dot, so the influence of the transported gas on the height and radius of the underwater gas film/bulge at the outlet dot is negligible. *H*_2_ and *R*_2_ could still be simplified to *H*_2_ ≈ 0 and *R*_2_ ≈ ∞ during gas transportation. The pressure difference (Equation (6)) could be approximately expressed as

(10) $$\Delta P\approx \frac{2\gamma }{R_{1}}-\rho gH_{1}=\frac{4H_{1}\gamma }{H_{1}^{2}+r_{1}^{2}}-\rho gH_{1}$$

The spontaneous gas transportation process driven by the pressure gradient between the underwater gases at the inlet and outlet dot depended on the pressure difference and the hydrodynamic resistance (*Z*) of the connecting microchannel. The volumetric flow rate (*Q*), the change in volume with respect to time, could be calculated using the following equation^[^ ^28^, ^31^, ^32^, ^43^ ^]^

(11) $$Q=-\frac{dV}{dt}=\frac{1}{Z}\left(\Delta P\right)$$

Therefore, the following differential equation could be obtained to describe the evolution of the gas bubble at the inlet dot during gas transportation

(12) $$-\pi \left(\frac{H_{1}^{2}+r_{1}^{2}}{2}\right)\frac{dH_{1}}{dt}=\frac{1}{Z}\left(\frac{4H_{1}\gamma }{H_{1}^{2}+r_{1}^{2}}-\rho gH_{1}\right)$$

This differential equation could be solved with MATLAB software. The unknown constant parameter *Z* was calculated from the experimental datum points. Combined with Equation (7), the variation in gas volume with time could be easily predicted for the gas transportation process, as shown in Figure 3g (Model‐1 line). The model values were in good agreement with the first half of the experimental measurements. The deviation of the second half of the experimental measurements was ascribed to the formation of the gas bulge at the outlet dot, which was slightly different from the approximation hypothesis.

When the outlet dot was comparable in area to the inlet dot, the influence of the shape of the gas bulge at the outlet dot on the transportation process could not be ignored. It was difficult to calculate the effect of the height and radius of the underwater gas film/bulge at the outlet dot directly because the gas bulge was irregular in shape on the square base. To simplify the model, it was approximately assumed that the gas bulge at the outlet dot had a spherical surface and that the bottom diameter of the gas bulge was roughly equal to the length of the sides of the square. This approximate treatment led to the following set of equations^[^ ^31^ ^]^

(13) $$\left\{\begin{array}{c} R_{2}=\frac{H_{2}^{2}+r_{2}^{2}}{2H_{2}}\\ V_{2}=\frac{\pi }{6}\left(H_{2}^{3}+3H_{2}r_{2}^{2}\right)=V_{0}-V=V_{0}-\frac{\pi }{6}\left(H_{1}^{3}+3H_{1}r_{1}^{2}\right) \end{array}\right.$$

where *r*_2_ is the radius of the bottom surface of the gas bulge, *V*_2_ is the volume of the gas bulge at the outlet dot, and *V*_0_ is the initial volume of the input gas at the inlet dot. In this case, the pressure difference could be deduced as^[^ ^28^, ^31^, ^32^, ^44^ ^]^

(14) $$\Delta P=P_{1}-P_{2}=4\gamma \left(\frac{H_{1}}{H_{1}^{2}+r_{1}^{2}}-\frac{H_{2}}{H_{2}^{2}+r_{2}^{2}}\right)-\rho g\left(H_{1}-H_{2}\right)$$

The volumetric flow rate also followed Equation (11). Therefore, the gas transportation process could be described by the following differential equation

(15) $$\begin{array}{ccc} & & -\pi \left(\frac{H_{1}^{2}+r_{1}^{2}}{2}\right)\frac{dH_{1}}{dt}\\ & & \;=\frac{1}{Z}\left(4\gamma \left(\frac{H_{1}}{H_{1}^{2}+r_{1}^{2}}-\frac{H_{2}}{H_{2}^{2}+r_{2}^{2}}\right)-\rho g\left(H_{1}-H_{2}\right)\right) \end{array}$$

From the set of differential equations (Equations (15), (13), and (7)), the gas volume change as a function of time could be calculated, as depicted in Figure 3g (Model‐2 line). The model line agreed well with the measured values, especially for the second half of the experimental measurements. The slight deviation mainly came from the difference between the spherical approximation and the actual situation.

### Statistical Analysis

The diameter of the input gas bubble at the inlet dot was smaller than the capillary length, so the gas bubble could be considered approximately spherical. Therefore, the gas volume at the inlet dot from the base diameter and height of the gas bubble based on the captured profile image was calculated. The volume of transferred gas was calculated by subtracting the volume of the gas remaining at the inlet dot from the initial input gas volume. The differential equations were solved with MATLAB software. The average values in this experiment were obtained by measuring five times.

## Conflict of Interest

The authors declare no conflict of interest.

## Supporting information

Supporting Information

Supplemental Movie 1

Supplemental Movie 2

Supplemental Movie 3

Supplemental Movie 4

Supplemental Movie 5

Supplemental Movie 6

Supplemental Movie 7

Supplemental Movie 8

Supplemental Movie 9

Supplemental Movie 10

Supplemental Movie 11

Supplemental Movie 12

Supplemental Movie 13

Supplemental Movie 14

Supplemental Movie 15

Supplemental Movie 16

## Data Availability

The data that support the findings of this study are available from the corresponding author upon reasonable request.
