# Supplementary material for: Self‐Driving Underwater “Aerofluidics”
Source: Adv Sci (Weinh). 2023 Apr 28;10(21):2301175. doi: 10.1002/advs.202301175 (PMC10375095; doi:10.1002/advs.202301175)
Supplement: Supplementary file 1 — Supporting Information [file ADVS-10-2301175-s006.pdf]

ADVANCED SCIENCE
Open Access

Supporting Information

for *Adv. Sci.*, DOI 10.1002/advs.202301175

Self-Driving Underwater "Aerofluidics"

*Jiale Yong, Yubin Peng, Xiuwen Wang, Jiawen Li, Yanlei Hu, Jiaru Chu and Dong Wu**

Supporting Information

Self-Driving Underwater "Aerofluidics"

*Jiale Yong, Yubin Peng, Xiuwen Wang, Jiawen Li, Yanlei Hu, Jiaru Chu, and Dong Wu**

CAS Key Laboratory of Mechanical Behavior and Design of Materials, Key Laboratory of Precision Scientific Instrumentation of Anhui Higher Education Institutes, Department of Precision Machinery and Precision Instrumentation, University of Science and Technology of China, Hefei, 230027, P. R. China

E-mail: dongwu@ustc.edu.cn

Including Movie S1-S16 and Figure S1-S21

Part A: Caption of the Supplementary Videos

Movie S1. Rolling and rebounding processes of water droplets on the laser-structured superhydrophobic surface.

Movie S2. Gas spreading out on the laser-structured superhydrophobic surface in water.

Movie S3. Self-driving gas transportation of the designed aerofluidic device in water.

Movie S4. Underwater gas transportation with continuously inputting bubbles into the aerofluidic system.

Movie S5. Underwater gas transportation of the aerofluidic device with different area ratios of the outlet to inlet dots.

Movie S6. Underwater gas transportation at different inclination angles.

Movie S7. Gas transportation along curved paths (continuous-curve line, spiral line, and curved surface).

Movie S8. Gas transportation capacity of the aerofluidic devices by gradually increasing the gas transportation length.

Movie S9. Volume change of gas with a transportation distance of 1 m.

Movie S10. Underwater gas transportation across different aerofluidic devices.

Movie S11. Stable gas transportation ability of the underwater aerofluidic devices in different harsh environments.

Movie S12. Self-driving gas transportation on different substrates (PTFE, Glass, Si, and Al).

Movie S13. Processes of gas merging and aggregation.

Movie S14. Process of the formation of a gas array.

Movie S15. Explosive microreaction between H_2 and O_2 gases on the aggregation-functional aerofluidic device.

Movie S16. Microreaction between the CO_2 gas and the alkaline NaOH solution consisting of phenolphthalein reagent on the underwater aerofluidic device.

Part B: Supplementary Figures

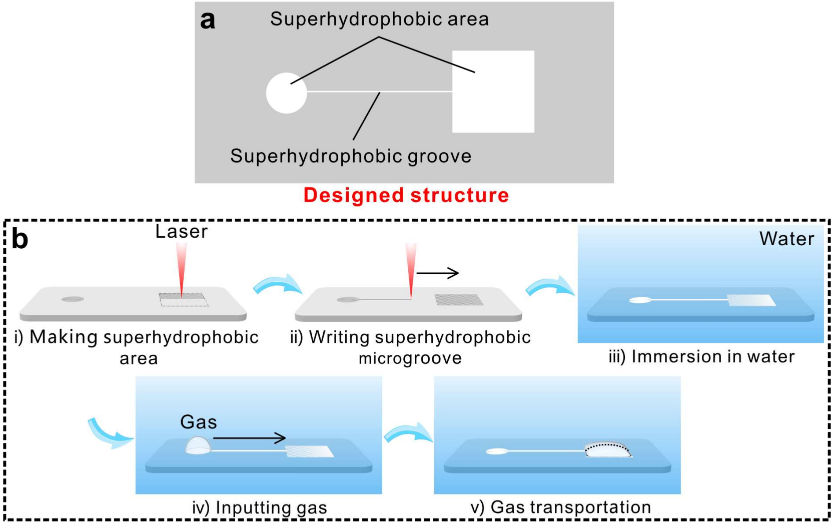

Figure S1. Gas transportation on the designed aerofluidic device. (a) The designed structure of the simplest underwater aerofluidic device in which a straight superhydrophobic microgroove connects two different superhydrophobic regions. (b) Schematic illustration of the verification process of gas transportation on the designed aerofluidic device, including five steps: (i) preparation of superhydrophobic regions (as the inlet dot and outlet dot) by femtosecond laser, (ii) femtosecond laser writing a superhydrophobic microgroove that connects the inlet dot and outlet dot, (iii) immersion of the as-prepared device in water, (iv) inputting gas onto the inlet dot, and (v) after gas transportation.

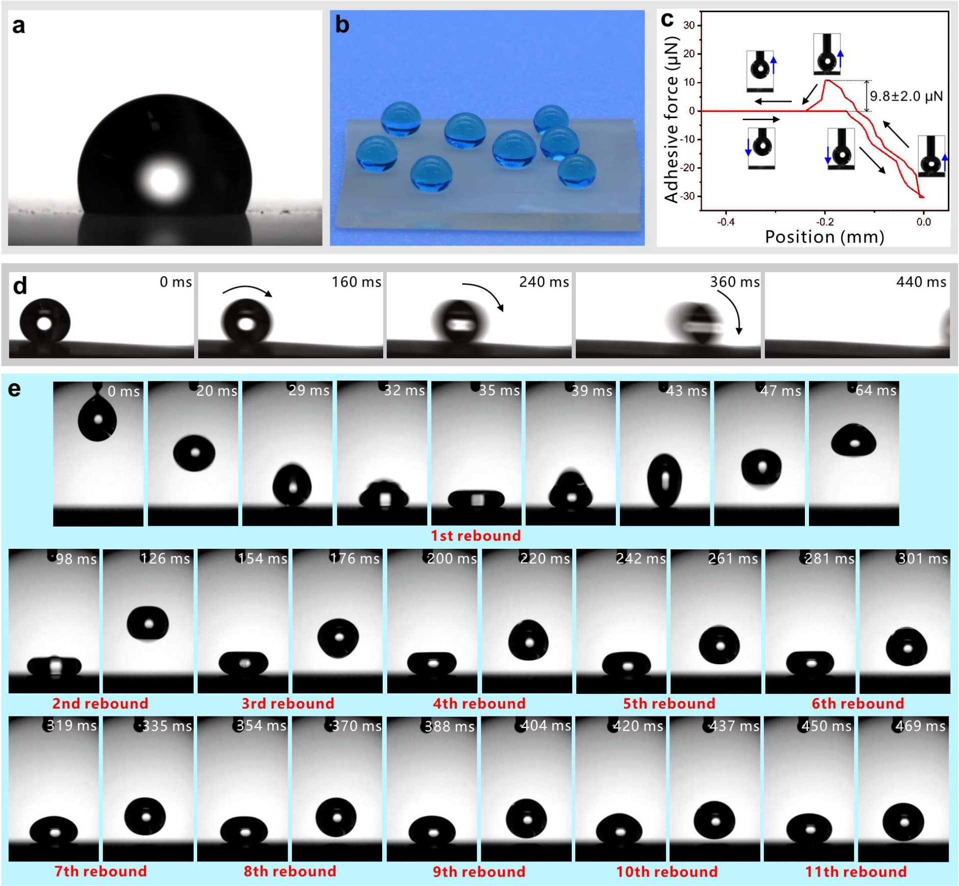

Figure S2. Superhydrophobicity of the femtosecond laser-structured PDMS surface. (a) Profile of a water droplet on the untreated flat PDMS surface. (b) Photograph of water droplets (dyed blue) on the superhydrophobic PDMS surface. (c) Adhesive force measurement between a water droplet and the laser-structured surface. (d) Process of a water droplet rolling on the laser-structured surface at a tilted angle of 1.4°. (e) The sequence of a free-falling water droplet impacting the laser-structured surface and rebounding more than 11 times.

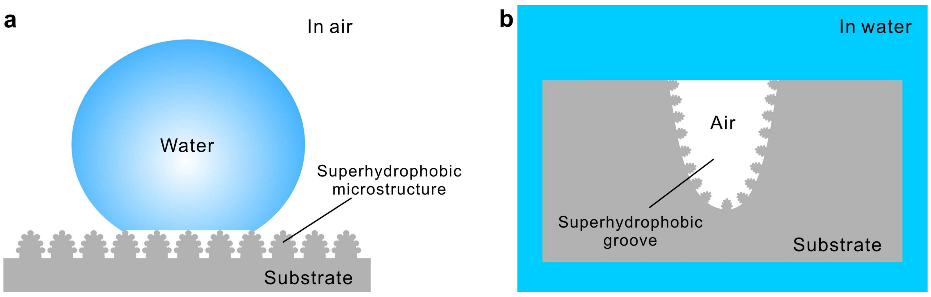

Figure S3. (a) Contact state between the water droplet and the femtosecond laser-ablated PDMS surface in air. Water only contacts with the top of the laser-induced superhydrophobic microstructure. (b) The wetting state between the laser-induced microgroove and water environment. A hollow microchannel forms between the microgroove and the water cover as water cannot penetrate the superhydrophobic microgroove.

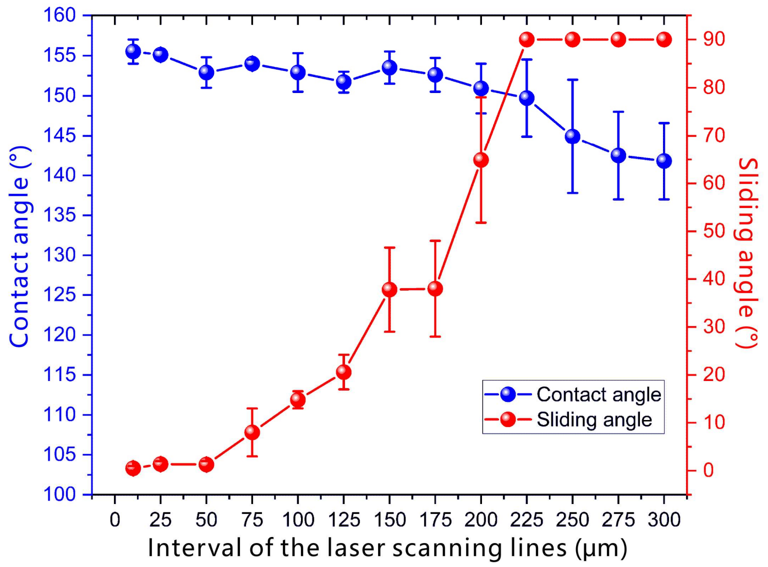

Figure S4. Influence of the interval (Λ) of the laser scanning lines on the wettability of the laser-treated surface. With the increase of Λ, the CA value of a water droplet on the laser-structured surface gradually decreases, while the SA value, as well as the adhesion to water, rises.

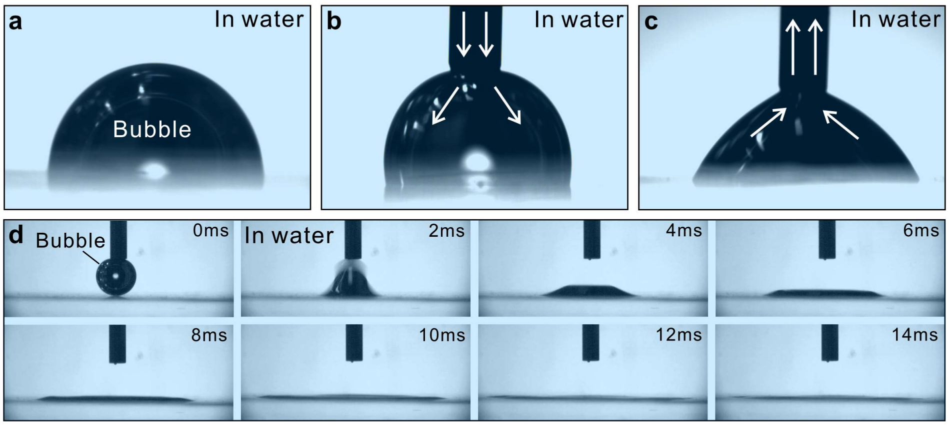

Figure S5. Wettability of gas bubble on the PDMS surface in water. (a) Profile of a bubble on the untreated flat PDMS surface. (b) Measurement of the advancing contact angle of the bubble on the flat PDMS surface by inputting gas. (c) Measurement of the receding contact angle of the bubble on the flat PDMS surface by extracting gas. (d) Process of an underwater bubble spreading out on the femtosecond laser-structured PDMS surface after the bubble contacting the sample surface.

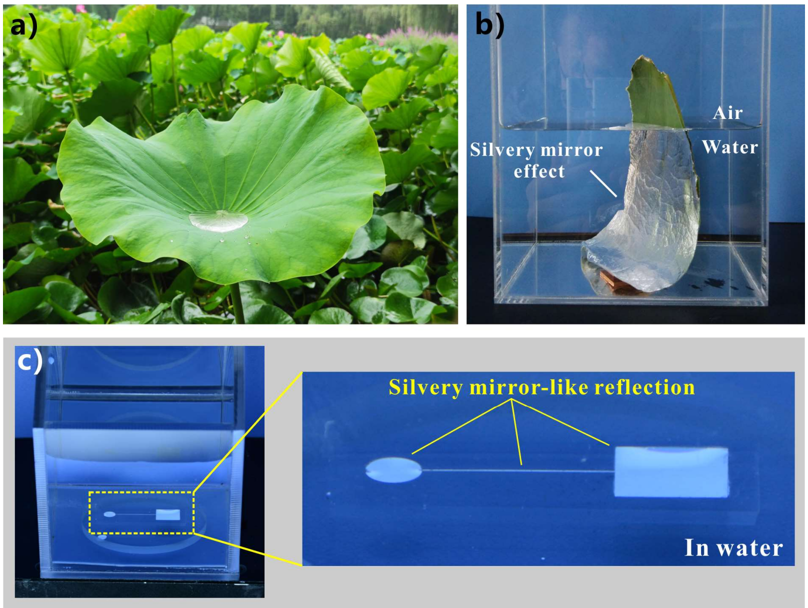

Figure S6. The silver mirror effect of the aerofluidic microstructure in water. (a) Photograph of a lotus leaf. (b) Immersion of a piece of a lotus leaf in water. The underwater part of the lotus leaf looks like a silver mirror. (c) Photograph of the as-prepared aerofluidic device in water. When the aerofluidic device is dipped into water, the superhydrophobic regions and the connecting line show mirror-like reflection because these areas are not wetted by water and air is trapped between superhydrophobic microstructures and the water environment.

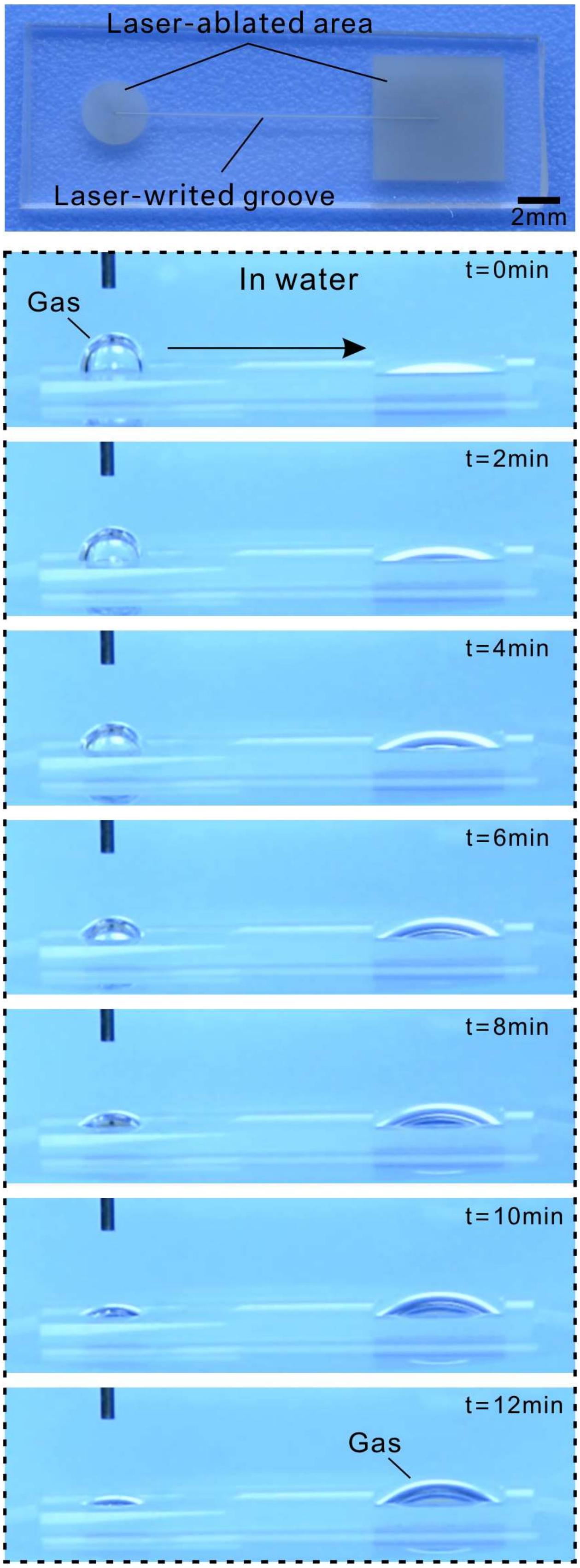

Figure S7. Time-lapse images of the whole process of self-driving gas transportation from the inlet to the outlet dot of the as-prepared aerofluidic device in water.

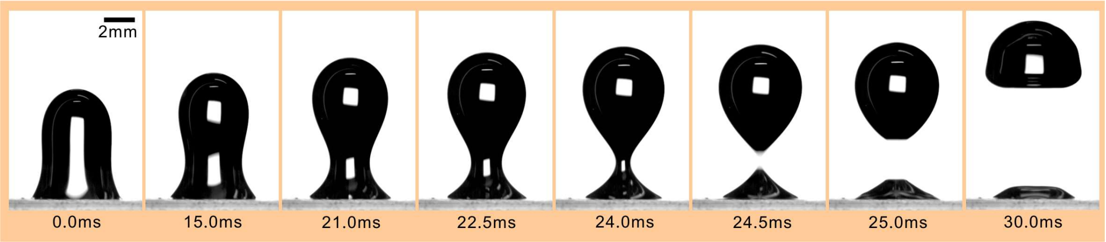

Figure S8. The process of the underwater gas bulge detaching from the outlet dot and rising to the water surface when its volume and buoyancy are large enough.

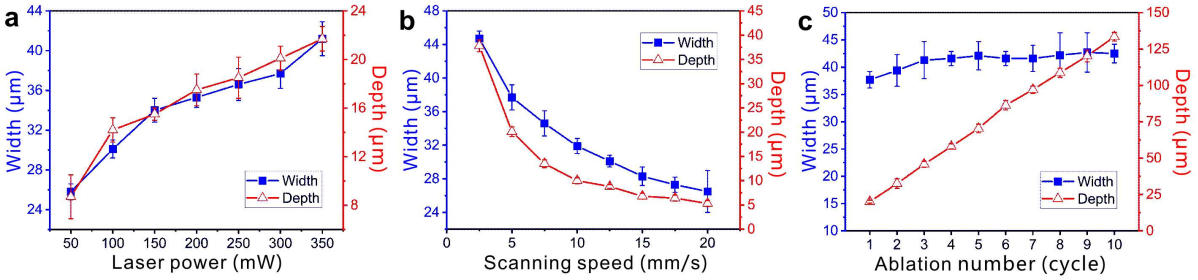

Figure S9. The influence of (a) the laser power, (b) the laser scanning speed, and (c) the ablation times on the width and depth of the resultant microgrooves.

Figure S9a shows the effect of laser power on the width and depth of the fabricated superhydrophobic microgroove. The results show that both the width and depth increase with increasing laser power. With higher laser energy, wider and deeper microgrooves can be obtained because higher laser power causes a more intense ablation process and more material removal. Different from the laser power, the width and depth are inversely correlated with the laser scanning speed, as shown in Figure S9b. The faster the scanning speed is, the less laser energy is accumulated per unit area, resulting in less material removal. As the scanning speed increases, the width and depth gradually decrease. Multiple repeated scanning can produce a deep microgroove. Figure S9c shows the width and depth of the laser-etched microgrooves when the focused laser scans the microgroove position for different times. The result indicates that the width is less affected by the number of ablation times, but the depth of the microgroove obviously increases with the increase of ablation times. In particular, the depth grows almost linearly with the number of ablations, so the depth of the microgrooves can be well-designed.

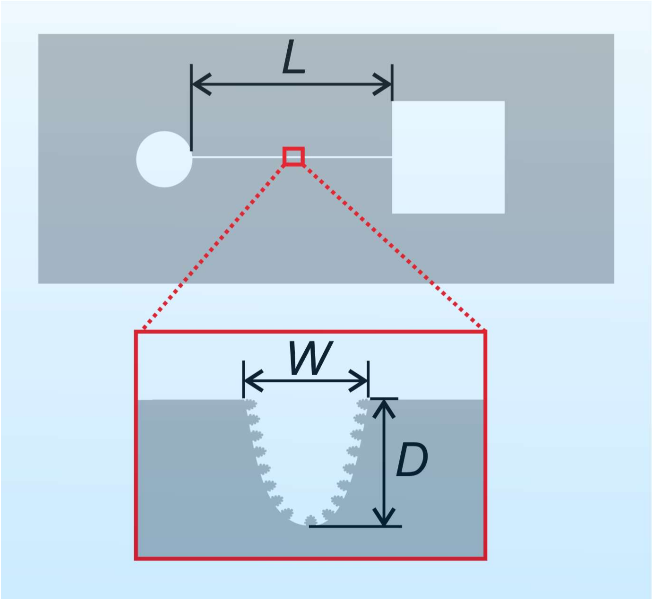

Figure S10. The description of length (*L*), depth (*D*), and width (*W*) of the laser-induced connecting microgroove of the as-prepared underwater aerofluidic device.

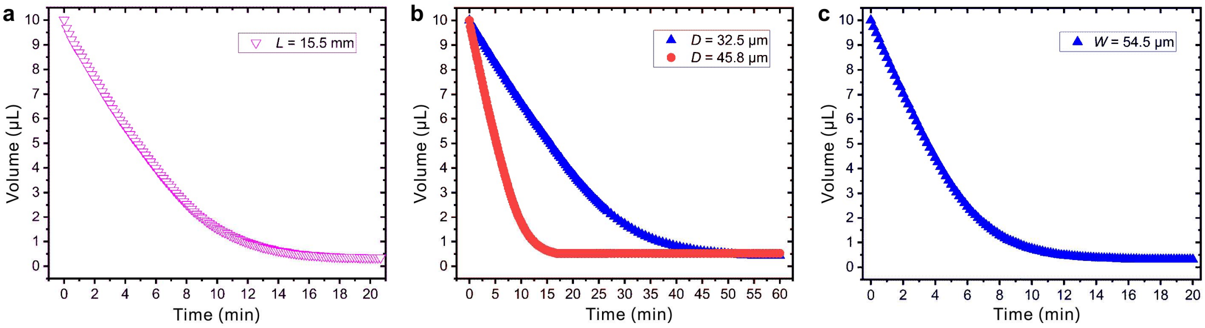

Figure S11. The complete trends of gas variation of (a) L =15.5 mm, (b) D = 32.5 μm and D = 45.8 μm, and (c) W = 54.5 μm corresponding to the data in Figure 4c-e.

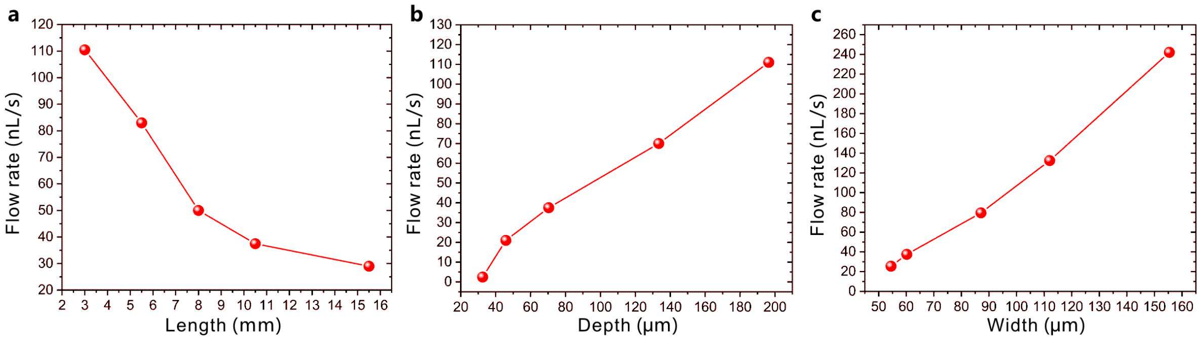

Figure S12. Influence of the (a) length, (b) depth, and (c) width of the laser-induced connecting microgrooves on the initial gas flow rate of the gas transportation process. The data in these figures corresponds to the data in Figure 4c-e.

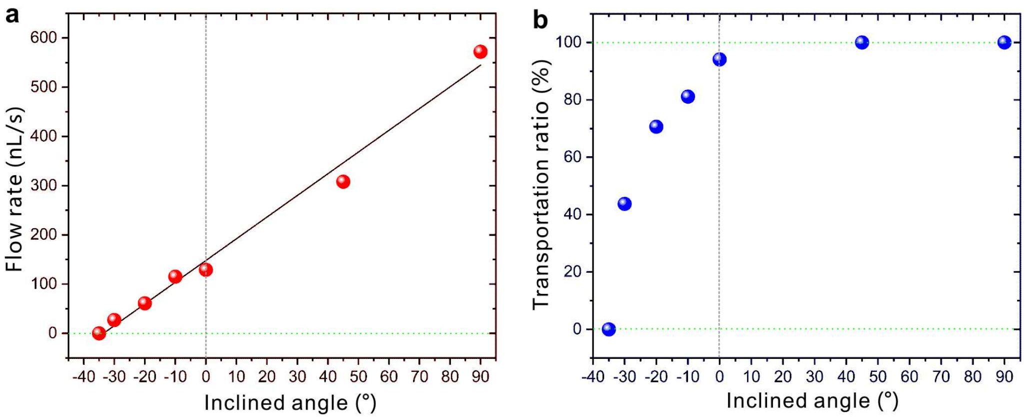

Figure S13. Influence of the inclination angle on the gas flow rate and the gas transportation ratio of the underwater aerofluidic device. (a) The initial gas flow rate of the aerofluidic device at different inclination angles. (b) Gas transportation ratio of the aerofluidic device at different inclination angles.

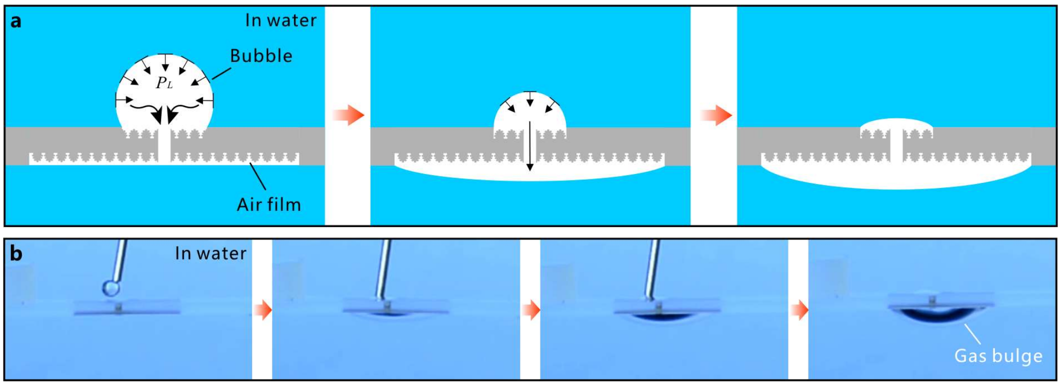

Figure S14. Anti-buoyance penetration process of gas through the PDMS sheet. (a) Schematic of the mechanism and driving force (Laplace pressure) of the gas transportation process from one side of the PDMS sheet to another in an aqueous environment. In this type of underwater aerofluidic device, the small inlet (upper) and large outlet (bottom) dots are separated on either side of the sheet and connected through a superhydrophobic through-hole. (b) Experimental process of anti-buoyance gas transportation with continuously inputting gas into the upper side of the device. When the gas is inputted onto the inlet dot of this device in water, the gas can transport from the upper surface of the PDMS sheet to the lower surface through the superhydrophobic through-hole.

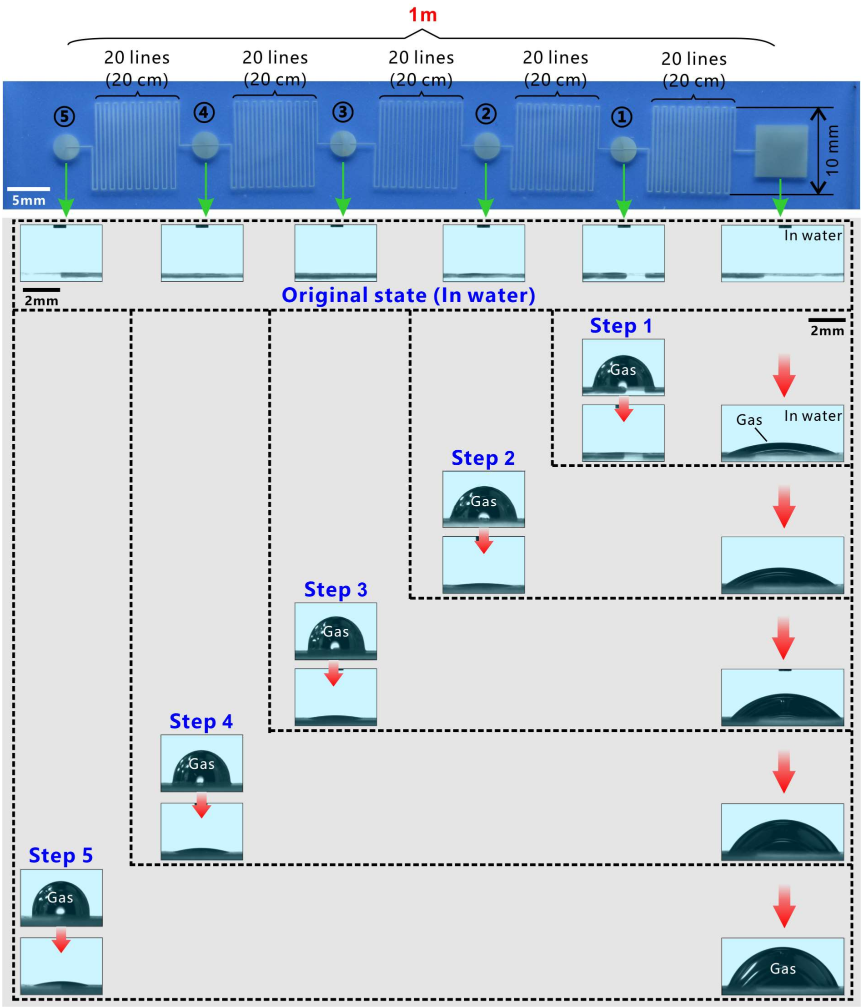

Figure S15. The measurement of the gas transportation capacity of the aerofluidic devices by gradually increasing the gas transportation length (connecting the superhydrophobic microgrooves in series).

In order to study the gas transportation capacity of the aerofluidic devices, we connect the superhydrophobic microgrooves in series, as shown in Figure S15. Each component contains a superhydrophobic curved microgroove with a length of 20 cm. When a 10 μL bubble is released to the No. 1 inlet dot of the integrated device, the volume of the bubble decreases over time. Meanwhile, the gas emerges and expands at the square outlet dot (Step 1). Finally, all the gas flows to the outlet dot. When a new bubble is further added to the No. 2 inlet dot, the gas in this bubble can also be transported to the outlet dot (Step 2). Similarly, when bubbles are gradually added at the No. 3 (Step 3), No. 4 (Step 4), and No. 5 (Step 5) inlet dots, it can be found that the bubbles in these areas disappear as the gas is transported away. At the same time, as more and more gas is successfully transported to the outlet dot, the volume of the gas bulge at the outlet dot keeps increasing. Even if the transportation distance is increased to more than 1 m, the input gas can also successfully flow from the inlet dot to the outlet dot, which indicates that the aerofluidic devices have an extremely long transportation capacity.

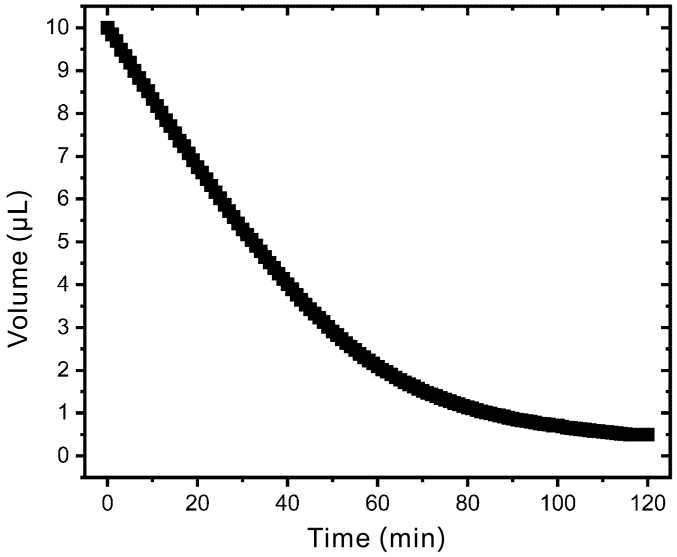

Figure S16. Volume change of the gas at the inlet dot with time during transportation. The integrated underwater aerofluidic device contains five curved-line components with a total length of microgrooves greater than 1 m.

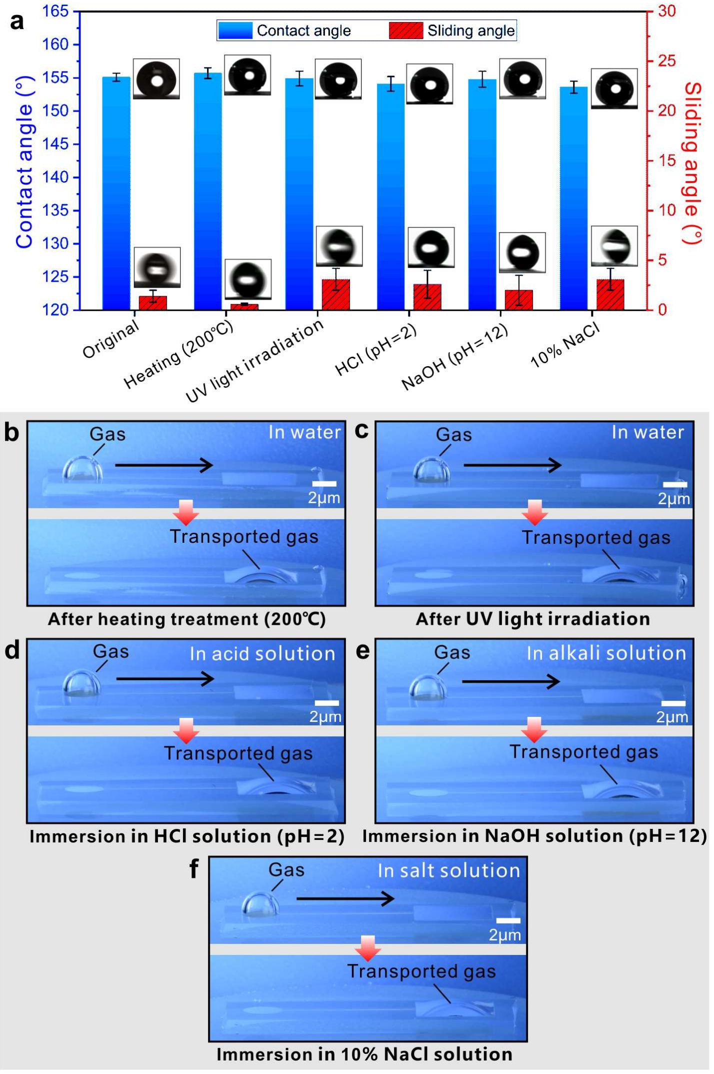

Figure S17. The durability of the femtosecond laser-induced superhydrophobic microstructures and the stable gas transportation ability of the underwater aerofluidic devices. (a) Wettability (including contact angle and sliding angle) of water droplets on the superhydrophobic PDMS surface after various damage treatments: heating at 200 °C for 6 h, UV light irradiation for 6 h, immersion in HCl solution (pH = 2) for 6 h, immersion in NaOH solution (pH = 2) for 6 h, and immersion in 10% NaCl solution for 6 h. (b,c) Underwater gas transportation of the aerofluidic device after (b) heating treatment and (c) UV light irradiation. (d-f) Gas transportation ability of the aerofluidic device in (d) HCl acid solution, (e) NaOH alkali solution, and (f) NaCl salt solution.

The laser-induced PDMS microstructures exhibit very stable superhydrophobicity. The surface can maintain great superhydrophobicity even if it suffers from various damage treatments, such as heating at 200 °C, UV light irradiation, immersion in HCl acid solution (pH = 2), immersion in NaOH alkali solution (pH = 12), and immersion in 10% NaCl salt solution for 6 h, respectively, as shown in Figure S17a. The stability of the superhydrophobicity allows the as-prepared aerofluidic device to work in a variety of harsh environments. Figure S17b and c show the underwater gas transportation process of the aerofluidic device that was previously heated at 200 °C and irradiated by UV light, respectively. Figure S17d-f shows the gas transportation process of the underwater aerofluidic device immersed in a strong acid solution, strong alkali solution, and high-concentration salt solution, respectively. The results demonstrate that the gas can normally be transported from the inlet dot to the outlet dot on the aerofluidic device regardless of whether the device is heated and irradiated by UV light or works in acid, alkali, and salt solutions.

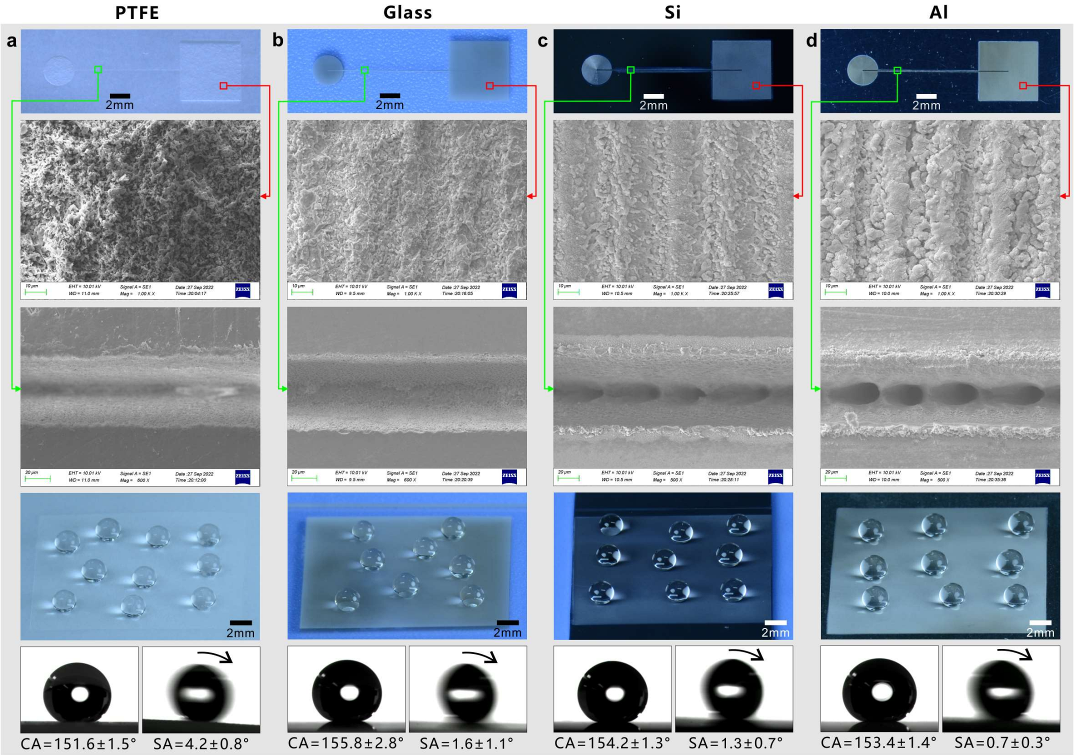

Figure S18. Morphology and superhydrophobicity of the femtosecond laser-induced microstructures on the (a) PTFE, (b) glass, (c) silicon, and (d) aluminum substrates. The first line shows the aerofluidic structures on these substrates. The second and third lines are the SEM images of the laser-induced microstructures and microgrooves on the as-prepared underwater aerofluidic devices. The fourth line shows the water droplets on the laser-induced microstructures of different substrates. The fifth line shows the profile (left images) of the water droplet on the laser-induced microstructures and the rolling process (right images) of the droplets on the slightly tilted surfaces.

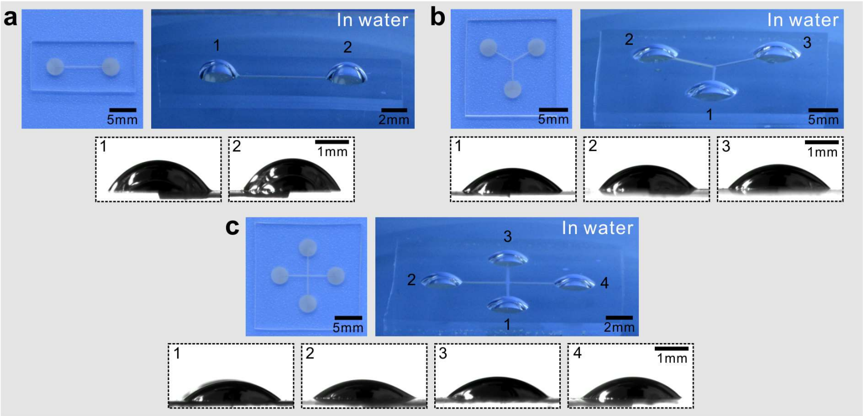

Figure S19. Splitting a gas bubble into (a) two, (b) three, and (c) four equal parts. The top-left images show the structures of the aerofluidic devices for gas equipartition, the top-right images show that a bubble is split equally in the circles, and the bottom insets are the profiles of the divided gas parts corresponding to the sequence in the top-right images.

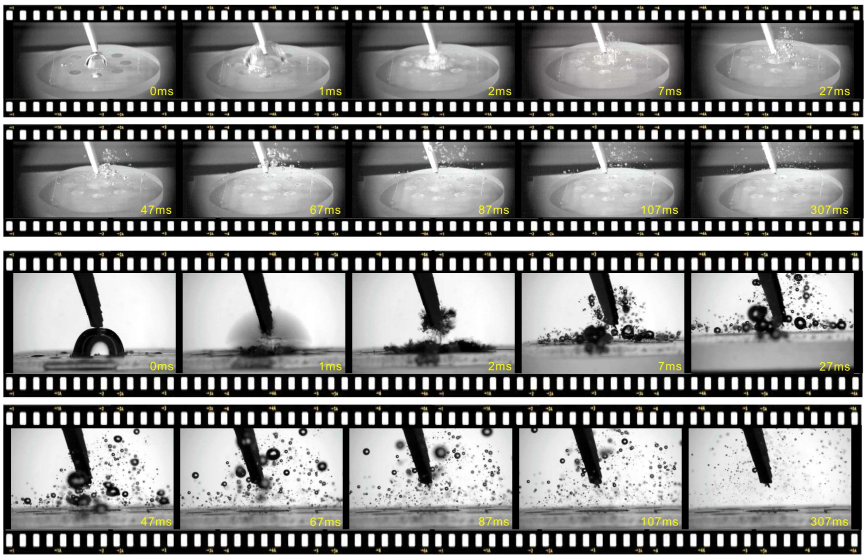

Figure S20. Explosive microreaction of the H_2 and O_2 mixing gas ignited by a piezoelectric ceramic electron flame. The top sequence is a downward oblique view, and the bottom sequence is a side view.

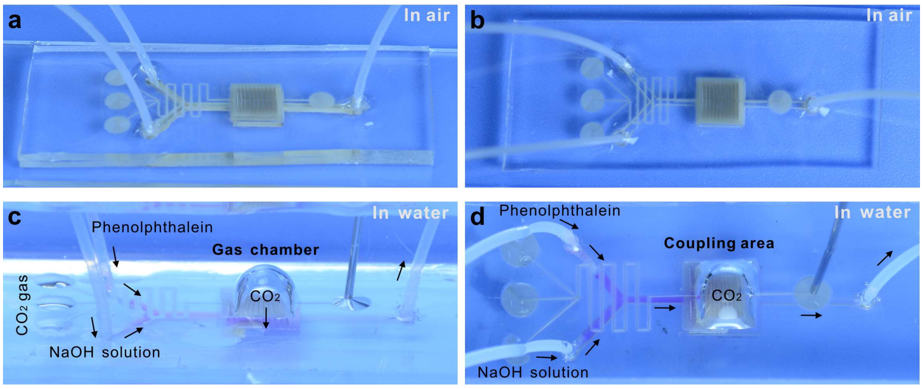

Figure S21. Integration of underwater aerofluidic chip and microfluidic chip. (a,b) Photograph of the coupling device: (a) downward oblique view and (b) top view. The upper part of the device is an aerofluidic surface structure, and the inner part is a simple liquid microfluidic system. (c,d) Proof-of-concept demonstration of the interaction between the upper underwater aerofluidic system and the internal microfluidic system of the coupling device: (c) downward oblique view and (d) top view.

NaOH solution and phenolphthalein solution are slowly injected into the microfluidic system. A reaction occurs when the two liquids meet, and the mixture appears purple. CO_2 gas is also input into the inlet dots of the underwater aerofluidic system, resulting in the formation of a gas chamber in the coupling zone. CO_2 gas can permeate through the aerofluidic chip along the superhydrophobic slit and make contact the mixed liquid flow in the microfluidic system. The reaction of the CO_2 gas with the mixed liquid causes the liquid flow in the microfluidic system to change from purple to transparent.
